# Ultrahigh-throughput single-pixel complex-field microscopy with frequency-comb acousto-optic coherent encoding (FACE)

**DOI:** 10.1038/s41377-025-01931-w

**Published:** 2025-08-11

**Authors:** Daixuan Wu, Yuecheng Shen, Zhongzheng Zhu, Tijian Li, Jiawei Luo, Zhengyang Wang, Jiaming Liang, Zhiling Zhang, Yunhua Yao, Dalong Qi, Lianzhong Deng, Zhenrong Sun, Meng Liu, Zhi-Chao Luo, Shian Zhang

**Affiliations:** 1 Guangdong Provincial Key Laboratory of Nanophotonic Functional Materials and Devices, Guangdong Basic Research Center of Excellence for Structure and Fundamental Interactions of Matter, School of Optoelectronic Science and Engineering, South China Normal University, Guangzhou, Guangdong 510006 China; 2 State Key Laboratory of Precision Spectroscopy, School of Physics and Electronic Science, East China Normal University, Shanghai, 200241 China; 3 School of Electronics and Information Technology, Sun Yat-sen University, Guangzhou, 510006 China; 4 MOE Key Laboratory of Laser Life Science, School of Optoelectronic Science and Engineering, South China Normal University, Guangzhou, 510631 China; 5 Joint Research Center of Light Manipulation Science and Photonic Integrated Chip of East China Normal University and Shandong Normal University, East China Normal University, Shanghai, 200241 China; 6 Collaborative Innovation Center of Extreme Optics, Shanxi University, Taiyuan, 030006 China

**Keywords:** Imaging and sensing, Microscopy, Biophotonics

## Abstract

Single-pixel imaging (SPI) is a promising technology for optical imaging beyond the visible spectrum, where commercial cameras are expensive or unavailable. However, limitations such as slow pattern projection rates and time-consuming reconstruction algorithms hinder its throughput for real-time imaging. Consequently, conventional SPI is inadequate for high-speed, high-resolution tasks. To address these challenges, we developed an ultrahigh-throughput single-pixel complex-field microscopy (SPCM) system utilizing frequency-comb acousto-optic coherent encoding (FACE). This system enables real-time complex-field monitoring in the non-visible domain. Operating at 1030 nm, our system achieves a record-high space-bandwidth-time product (SBP-T) of 1.3 × 10^7^, surpassing previous SPCM (~10^4^), SPI (~10^5^), and even certain types of commercial near-infrared cameras (~10^6^). It supports real-time streaming at 1000 Hz with a frame size of 80 × 81 pixels and a lateral resolution of 3.76 μm across an approximately 300 μm field of view. We validated the system by imaging dynamic transparent scenes, including microfluidics, live microorganisms, chemical reactions, as well as imaging through scattering media. This advancement offers a superior solution for high-speed, high-resolution complex-field imaging beyond the visible spectrum, significantly enhancing SPI performance across various applications.

## Abstract (web summary)

Given the ultrafast spatiotemporal nature of frequency-comb acousto-optic coherent encoding, ultrahigh-throughput single-pixel complex-field microscopy with a record-high space-bandwidth-time product enables real-time monitoring over various transparent dynamic scenarios in special spectrums.

## Graphical abstract

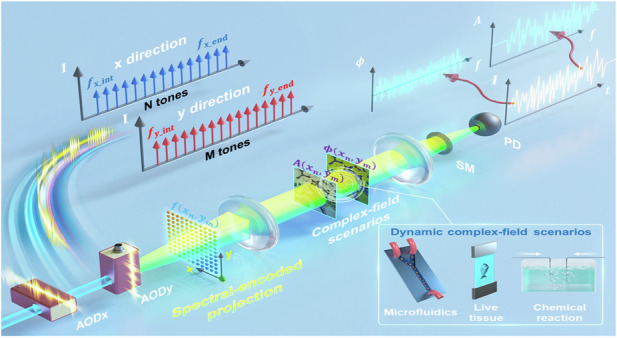

## Introduction

Optical imaging in the visible spectrum has advanced rapidly in recent years, largely due to mature silicon-based semiconductor fabrication technologies that enable high-performance cameras. However, for spectra beyond the visible range—such as near-infrared (NIR), X-ray, and terahertz—the development of pixel array detectors is still in its early stages. As a result, cameras operating in these non-visible spectra are often inefficient or prohibitively expensive, presenting significant challenges and costs for imaging in these domains. Despite these limitations, imaging in non-visible spectra is essential because it provides information that is often inaccessible with visible light, offering deeper insights into the structural and functional properties of various targets. For example, NIR imaging optimizes penetration depth and resolution while being sensitive to endogenous biological contrast agents like fat or protein^1,2^. This sensitivity enables the detection of subtle phenomena across biological, material, and chemical fields, driving advances in medical diagnostics, materials science, and chemical analysis^3–5^.

Single-pixel detectors provide superior performance across a broad spectral range compared to pixel-array detectors, driving advancements in optical imaging beyond the visible spectrum through an emerging computational technique known as single-pixel imaging (SPI)^6–10^. SPI utilizes the high detection capabilities of single-pixel detectors by combining active patterned illumination with computational image reconstruction^11–19^, making it a promising alternative for imaging in challenging spectral regimes such as mid-infrared^20,21^, terahertz^22–24^, and even photoacoustic waves^25,26^. Furthermore, SPI has been demonstrated to be extended to single-pixel complex-field microscopy (SPCM) within a holographic framework^27–29^, enabling the retrieval of both phase and amplitude information. This capability is particularly useful for observing dynamic phenomena in the NIR range, such as live cells, swimming microorganisms, and chemical reactions, which are difficult to differentiate using intensity-only images due to the relatively uniform absorption of biological samples and solutions^30^. However, SPI and SPCM systems typically have lower information throughput than pixel-array camera systems because they rely on a single detector for data collection, making real-time monitoring of rapidly changing dynamic scenes challenging. The throughput of these systems is determined by two key factors: frames per second (FPS) and frame size. The interplay between these factors necessitates a more comprehensive metric: the space-bandwidth-time product (SBP-T), which quantifies the amount of information collected per unit time by multiplying FPS and frame size^31,32^.

The primary limitation of the SBP-T in SPI/SPCM systems arises from slow pattern projection rates. While fast digital micromirror devices (DMDs) with projection rates up to 23 kHz have allowed SPI/SPCM systems to achieve SBP-T values between 10^3^ and 10^4 29,33–36^, further advancements are needed. Incorporating heterodyne holography has enabled a DMD-based SPCM system to achieve a fourfold increase in SBP-T, reaching 4.6 × 10^4^ ^37^. To further enhance SBP-T, overcoming the projection rate limitations of DMDs is essential. Researchers have developed methods to achieve higher pattern projection rates. One approach involves using a rotational mask pattern, which raises the projection rate to 2.4 MHz^38^. More advanced techniques have pushed projection rates to 14.1 MHz by combining laser-scanning hardware with a DMD^39^. However, high-speed mechanical scanning introduces instability, preventing these systems from consistently operating at their maximum projection rate limits. Moreover, higher projection rates increase the computational burden for image reconstruction, limiting FPS and reducing information throughput during real-time monitoring. For example, an SPI system using a rotational mask pattern achieves real-time imaging at 72 FPS with a frame size of 101 × 103 pixels, resulting in an SBP-T of 7.5 × 10^5^ ^38^. Another SPI system that combines laser-scanning hardware with a DMD achieves 51 FPS with a frame size of 59 × 61 pixels, yielding an SBP-T of approximately 1.8 × 10^5 39^. Despite these advancements, the SBP-T of SPI/SPCM systems remains several orders of magnitude lower than that of a commercial InGaAs camera operating in the NIR spectrum (e.g., ARTCAM-008TNIR, ARTRAY), which achieves an SBP-T of 7.4 × 10^6^ with a frame size of 320 × 256 and an FPS of 90 Hz.

To achieve SPI/SPCM throughput comparable to, or surpassing, that of commercial cameras in specialized spectra such as the NIR spectrum, it is crucial to develop innovative fast pattern projection schemes combined with efficient image reconstruction algorithms for streamlined real-time imaging. In this study, we present an ultrahigh-throughput SPCM system using frequency-comb acousto-optic coherent encoding (FACE), which outperforms commercial cameras in the NIR spectral regime. The FACE scheme achieves remarkable efficiency by integrating advancements in motionless fast pattern refresh with effective complex-field imaging reconstruction. For pattern refresh, the FACE scheme modulates projection patterns into two-dimensional frequency combs. The asynchronous oscillation of different tones within these combs allows the patterns to evolve rapidly over time, ensuring fast and stable pattern refresh without mechanical motion. For complex-field imaging reconstruction, the FACE scheme uses fast Fourier transformation (FFT) to accurately decode the spatial information of each tone, enabling efficient reconstruction without requiring extensive computational resources. Furthermore, the scheme maintains a zero-order unmodulated component as a coaxial reference, allowing for stable complex-field retrieval within a highly efficient common-path heterodyne holography setup.

The developed FACE-SPCM system enables real-time monitoring with a frame size of 80 × 81 at 1000 Hz, supporting complex-field microscopy with a lateral resolution of 3.76 μm and a field of view (FoV) of approximately 300 μm. Data acquisition and image reconstruction are performed efficiently, achieving a record-high SBP-T of up to 1.3 × 10^7^ in the NIR spectral regime. A comprehensive comparison of representative aforementioned traditional SPI/SPCM systems, along with a broader category of imaging systems, highlighting key imaging parameters, is provided in Supplementary Note 1. The SBP-T (~10^7^) achieved in this study is two to three orders of magnitude higher than previous records for SPCM (~10^4^) and SPI (~10^5^), and even surpasses those of certain commercial NIR cameras (~10^6^). A series of imaging experiments are conducted for various dynamic transparent scenes even through scattering media, including high-speed microfluidics, live microorganisms, and chemical reactions, demonstrating the capability of real-time diagnosis of physicochemical phenomena. This development paves the way for future imaging systems based on single-pixel detectors, establishing the FACE scheme as a leading solution for high-speed, high-resolution complex-field imaging.

## Results

### Operational principle of FACE-SPCM

The foundational concept for achieving ultrahigh-throughput SPCM is to use frequency components to encode spatial positions. This approach is similarly applied in various imaging fields that use different types of spectral encodings, such as time-stretch techniques in ultrafast optics and acousto-optic modulation for intensity oscillation scanning^40–45^. These methods, which primarily function within one-dimensional (1D) intensity scanning frameworks, have been tailored for specific applications like scanning-based flow cytometry and cell sorting^46–48^. Historically, a virtually imaged phased array combined with a grating has been used to create a two-dimensional (2D) mapping between frequency components and spatial positions, although this configuration has been limited by frame size^40,49^.

**Fig. 1:**
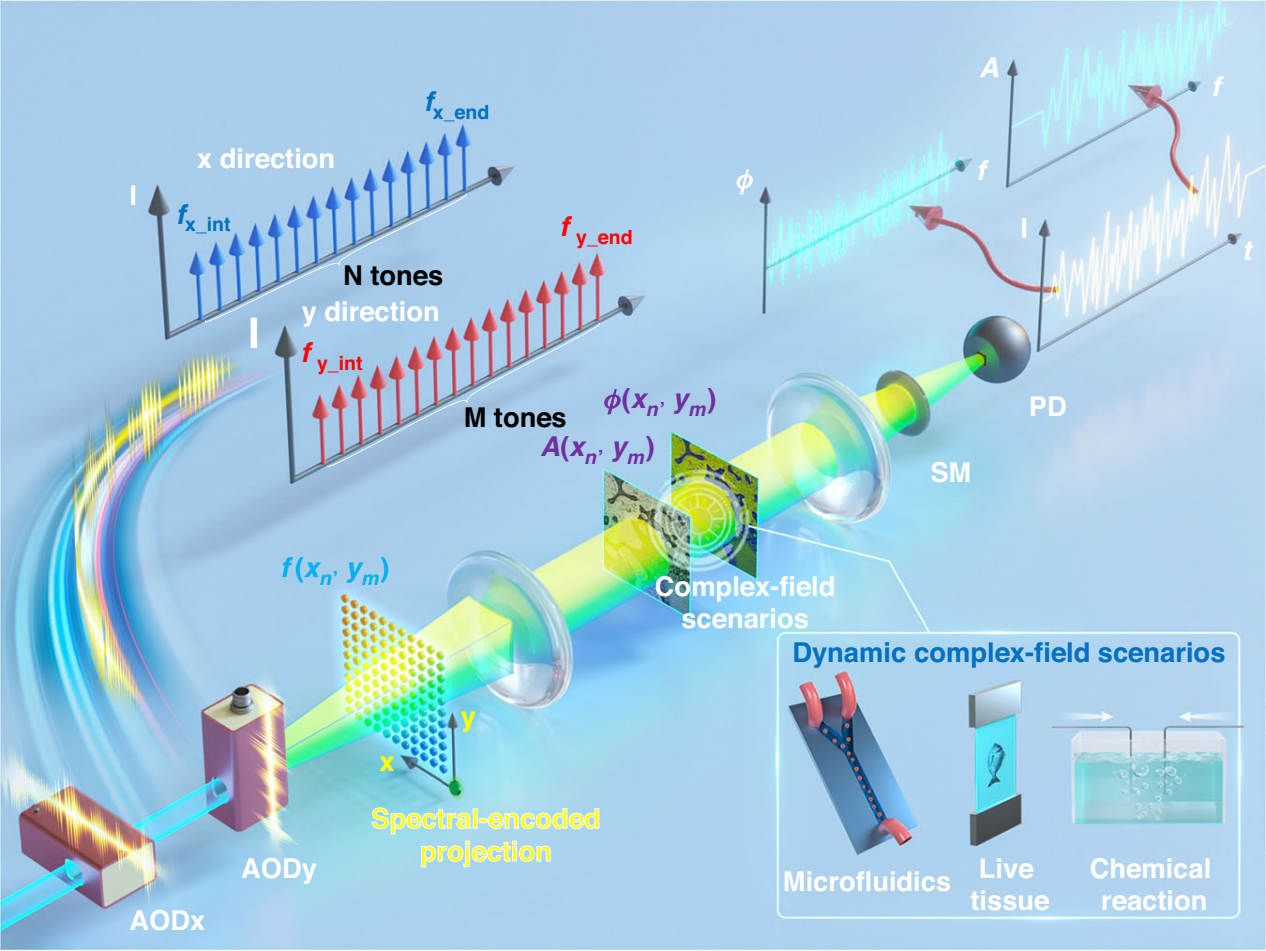
Schematic diagram of the FACE-SPCM system. The 2D spectral-encoded projection is generated by a pair of orthogonal optical frequency combs through acousto-optic modulation. Complex-field details of the scene are encoded within a temporal sequence using a coaxial heterodyne holographic setup. This configuration enables the efficient retrieval of complex-field information in various dynamic scenarios, such as microfluidics, live tissue, and chemical reactions. AODx (AODy): acousto-optic deflector in the *x* (*y*) direction; SM scattering medium, PD single-pixel photodetector

In this study, we extend the concept of spectral-encoded imaging to a more general 2D imaging scenario in complex fields by developing the FACE-SPCM system. The operational principle is illustrated in Fig. 1. This spectral-encoded projection scheme eliminates the need for time-consuming sequential projection of different illumination patterns by generating a pattern that evolves naturally over time. The key to extending spectral-spatial mapping from 1D to 2D with spectral-encoded projection is the use of a pair of cascaded acousto-optic deflectors (AODs) oriented in orthogonal directions. Each AOD is driven by different 1D frequency-comb electronic signals over the same modulation bandwidth of ${f}_{{\rm{range}}}={f}_{(x/y){\_}{\rm{end}}}-{f}_{(x/y){{\_}}\mathrm{int}}$, with *N* and *M* tones, respectively, and varied spacing between comb teeth of $\Delta{f}_{x}$ and $\Delta{f}_{y}$. Precise alignment of these AODs using a 4*f* system with identical or cylindrical lenses, as detailed in Supplementary Note 2, is crucial. To effectively demonstrate the FACE scheme for subsequent applications, the polarization conditions involved in spectral-encoded projection are detailed in Supplementary Note 3. Under these conditions, the FACE scheme enables a one-to-one mapping between spatial positions and frequency components, forming the basis of the ultrahigh-throughput SPCM system. A similar strategy has been employed for real-time wavefront shaping through scattering media using acousto-optic modulators with few degrees of freedom^50,51^.

Mathematically, the correspondence between spatial positions and frequencies in the FACE scheme is defined by:

1 $$f\left({x}_{n},{y}_{m}\right)={f}_{0}-{f}_{x}\left({x}_{n}\right)+{f}_{y}\left({y}_{m}\right)$$

where ${x}_{n}=\left(n-1\right)\Delta x,{y}_{m}=\left(m-1\right)\Delta y$, with $1\le n\le N,1\le m\le M$. Here, ${x}_{n}$ and ${y}_{m}$ represent the relative lateral positions in orthogonal directions, while ${f}_{0}$ is the base frequency of light at the reference point. $\Delta{x}$ and $\Delta{y}$ are the relative distances resulting from 2D Bragg diffraction along orthogonal directions. A detailed explanation for designing this FACE framework in coaxial heterodyne holography without overlap is provided in Supplementary Note 4.

As a 2D FACE pattern, the spectral-encoded projection interacts with the target object to achieve complex-field microscopy. For simplicity, while maintaining generality, we represent the complex-field information of the object as $O\left({x}_{n},{y}_{m}\right)=A\left({x}_{n},{y}_{m}\right)\exp \left(i\phi \left({x}_{n},{y}_{m}\right)\right)$, where $A\left({x}_{n},{y}_{m}\right)$ and $\phi \left({x}_{n},{y}_{m}\right)$ denote the 2D amplitude and phase distributions, respectively. After interaction, the resultant light field is described by:

2 $$E\left({x}_{n},{y}_{m},t\right)=O\left({x}_{n},{y}_{m}\right)\exp \left(i2\pi f\left({x}_{n},{y}_{m}\right)t\right)$$

Here, we assume each frequency component in the FACE pattern has equal amplitudes and phases. However, in practice, factors such as non-uniform circuit amplification, varying diffraction efficiencies, and differences in optical path lengths can cause significant variations in amplitude and phase. Nevertheless, in a linear imaging system, these effects can be compensated for through a null calibration process, as detailed in Supplementary Note 5.

To retrieve the phase information of the object, the FACE scheme is designed to maintain an unmodulated component with a frequency ${f}_{0}$ as a coaxial reference using a convergence grating, offering compact and robust complex-field imaging without external disturbances. To achieve high spatial coherence in coaxial interference, the optimal parameters for the convergence grating are detailed in Supplementary Note 6. In this configuration, the light detected by a single-pixel detector is given by:

3 $$I\left(t\right)={\left|\mathop{\sum }\limits_{n,m}O\left({x}_{n},{y}_{m}\right)\exp \left(i2\pi f\left({x}_{n},{y}_{m}\right)t\right)+{E}_{{\rm{R}}}\exp \left(i2\pi {f}_{0}t\right)\right|}^{2}$$

Here, ${E}_{{\rm{R}}}$ is the amplitude of the unmodulated component, i.e., the coaxially propagated reference light, which is significantly stronger than the amplitudes of other frequency components. The wide bandwidth of single-pixel detectors, which can reach hundreds of megahertz or even gigahertz, allows for efficient extraction of $O\left({x}_{n},{y}_{m}\right)$ through FFT within the holographic framework, enabling real-time data processing. A detailed reconstruction of SPCM for dynamic scenes using Fourier analysis is further elaborated in the “Methods” section. The digital flexibility of electronic signal modulation and the choice of physical equipment allow the FACE scheme to support various configurations, adaptable to different dynamic scenarios, as outlined in Supplementary Note 7. To show the superior scalability of FACE-SPCM, further demonstrations adopting alternative imaging schemes and imaging through scattering media are presented in Supplementary Notes 8 and 9.

### Characterization of imaging resolutions of the FACE-SPCM system

**Fig. 2:**
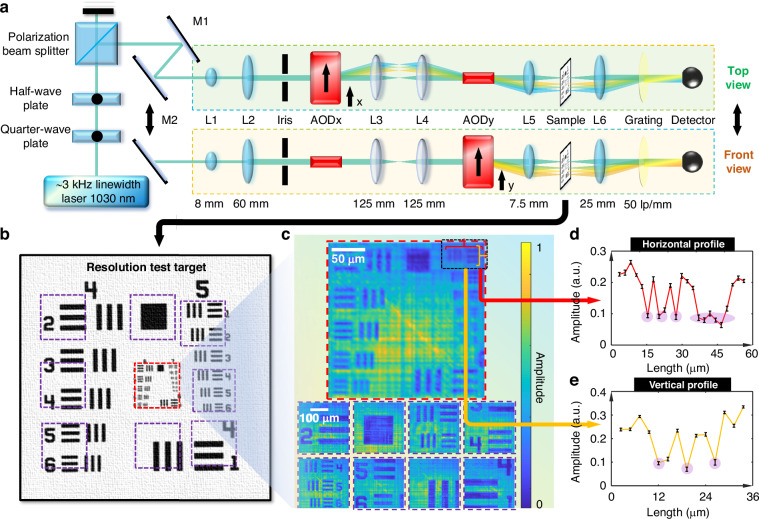
Experimental setup and imaging performance of the FACE-SPCM system. **a** The optical structure of the FACE-SPCM system is shown from both top and front views, with a resolution test target used for the initial demonstration of the FACE scheme. M1–M2: mirrors; L1–L6: lenses. **b** Cross-sectional diagram of the resolution test target, with dashed boxes in purple and red indicating the regions selected for imaging. **c** Reconstructed amplitude images corresponding to the marked regions are presented. The image framed in red (scale bar: 50 μm) shows the finest resolution lines, while images framed in purple (scale bar: 100 μm) demonstrate general imaging performance. Average CNR: 28.77. **d**, **e** Expanded 1D profiles from the highlighted area in (**c**), verifying the resolution capability of the FACE-SPCM system. **d** Horizontal profile. **e** Vertical profile. Error bar: standard deviations of five independent realizations

With the operational principle established, we constructed an experimental setup for SPCM integrated with the FACE system, as shown in Fig. 2a. For a comprehensive characterization of the FACE-SPCM system, full details of the experimental setup and its related parameters are provided in the “Methods” section. Rigorous characterization of the laser’s performance, particularly concerning spectral aliasing—critical to the integrity of the FACE-SPCM system—is thoroughly evaluated in Supplementary Note 10.

Our experimental demonstration began with a quantitative assessment of the imaging resolution. We used a standard positive resolution target (R3L3S1P, Thorlabs) as a benchmark to evaluate the system’s performance. Resolution is a critical parameter in SPCM systems as it directly impacts the ability to discern fine details. For our demonstration, the system was configured to achieve an effective frame size of 80 × 81, providing a lateral resolution of 3.76 μm across a FoV of approximately 300 μm. This setup enabled the system to resolve intricate structural details of the target, specifically those in group 7-1, which measure 3.91 μm. For simplicity, we focused on amplitude images, as the resolution profiles for both amplitude and phase imaging are identical in our system. To quantitatively assess imaging quality, we employed the contrast-to-noise ratio (CNR) rather than any reference-based metrics to evaluate the performance during practical experiments. This metric is defined as the difference in signal intensity between a target and the background region, divided by the background fluctuations, i.e., noise, in the reconstructed amplitude images. Figure 2b shows a high-definition image of the central portion of the resolution target, with dashed boxes indicating areas selected for detailed observation. The imaging results, presented in Fig. 2c, consist of nine separate images corresponding to predefined positions within Fig. 2b. A detailed analysis of the group 7-1 structure, centered in these images, was performed using 1D amplitude profiles plotted along the horizontal and vertical directions (Fig. 2d, e). These profiles clearly reveal three narrowly spaced dips and a single broader dip, confirming the system’s theoretical resolution capabilities.

### FACE-SPCM for microfluidics microscopy through scattering media

**Fig. 3:**
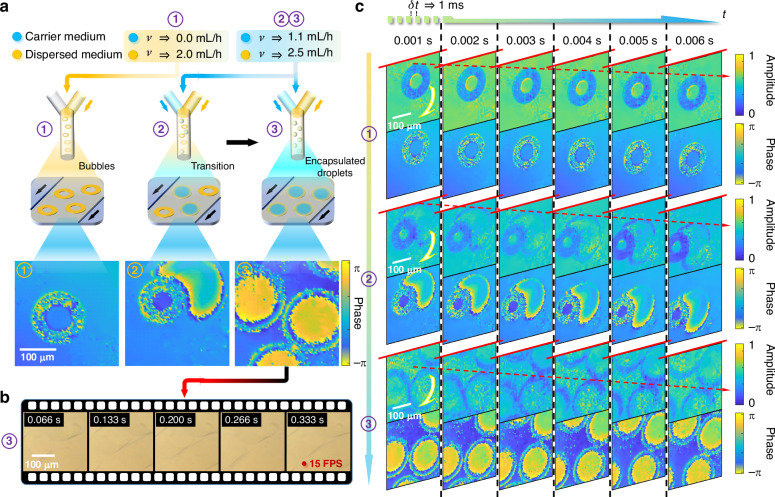
Experimental demonstration of FACE-SPCM in microfluidics microscopy. **a** The microfluidic device is designed for rapidly forming a sequence of flowing droplets through a microchannel. Water is used as the dispersed medium, while oil acts as the carrier medium. By varying the flow rates controlled by an automatic syringe pump, the system transitions through different states are clearly captured by FACE-SPCM, from bubbles to stable encapsulated droplets: (1) Stable bubble formation, (2) Transition from bubbles to encapsulated droplets, and (3) Stable encapsulated droplets. Scale bar: 100 μm. **b** Intensity microfluidics images obtained by bright-field microscope without phase revelation, captured in real time with a refresh rate of 15 FPS. Scale bar: 100 μm. **c** Reconstructed complex-field microscopy images of microfluidics captured in real-time FACE-SPCM with an ultrashort temporal interval of 1 ms. Scale bar: 100 μm. Average CNR: 16.55

We demonstrated the imaging capability of FACE-SPCM for microfluidics microscopy. The microfluidic setup is shown in Supplementary Note 11, where oil-encapsulated droplets are sequentially generated as they merge and flow through a channel in the chip. Details on the preparation of microfluidic chips for droplet formation are provided in sequence. The oil, serving as the carrier medium, dynamically interacted with various solutions, with pure water typically used as the dispersed medium. The interactions were precisely controlled by automated syringe pumps that regulate the flow rates, allowing the exploration of different fluidic behaviors and droplet dynamics. As displayed in Fig. 3a, varying flow rates controlled by automated syringe pumps cause transitions from bubble formation to encapsulated droplets, categorized as fluidic situations 1–3. Corresponding images for respective situations are also provided. However, due to their inability to provide phase information and relatively low FPS, conventional bright-field microscopes (such as the Mshot MSX2) are unable to perform real-time monitoring of high-speed transparent microfluidics, as evidenced by the blurred images shown in Fig. 3b. A microscopy video, slowed down by a factor of eight, is provided in Supplementary Movie S1.

The FACE-SPCM system, maintaining the high-resolution setting from earlier experiments, achieves complex-field microscopy with an imaging FPS of up to 1000 Hz. The dynamics of droplet formation were meticulously captured in a series of holograms, sampled at regular intervals, with various droplet morphologies showcased in Fig. 3c. This rapid sampling capability enables the observation of subtle flow variations within exceptionally brief time frames, with critical flowing changes highlighted by red dashed arrows. In fluidic situation 1, shown in Fig. 3c, the droplet formation process is depicted with the syringe pump for the carrier medium set at 2.0 mL h^−1^, while the dispersed medium is set at 0 mL h^−1^. Under these conditions, stable oil-encapsulated water droplets failed to form, instead resulting in bubbles that indicate unsteady flow dynamics. Although both oil and water are transparent with low absorption, their distinct optical properties—highlighted by their differential refractive indices—reveal the suboptimal conditions for stable droplet formation. In contrast, fluidic situations 2 and 3 in Fig. 3c show more favorable conditions with the syringe pump settings adjusted to 2.5 mL h^−1^ for the carrier medium and 1.1 mL h^−1^ for the dispersed medium. These adjustments clearly promote more stable droplet formation, as detailed in the comparative analysis. The corresponding dynamic video results, slowed down by eight times, are provided in Supplementary Movie S2. For a detailed exploration of dynamic imaging across different modalities, see Supplementary Notes 7 and 8. To verify the superior robustness against challenges, the FACE-SPCM system enables microfluidics microscopy through scattering media while consistently maintaining its FoV and resolution. The imaging results are provided in Supplementary Note 9, while dynamic video slowed down by eight times in Supplementary Movie S3, suggesting the potential applications of SPCM in biomedical and environmental sciences. It is important to note that in this study, the scattering medium is placed in front of the detector, following the same imaging configuration as previous works^52,53^. A more realistic non-invasive imaging scenario, where scattering occurs both before and after the target scene, remains unachievable using current SPI techniques.

To enhance the technical demonstration for biological applications, we also employed the FACE-SPCM system to image a group of paramecia for microorganism observation, with experimental results detailed in Supplementary Note 12. A corresponding video, slowed down by eight times, is included in Supplementary Movie S4. The preparation process is further elaborated in the “Methods” section.

### FACE-SPCM for real-time imaging of chemical reactions and solution mixing

**Fig. 4:**
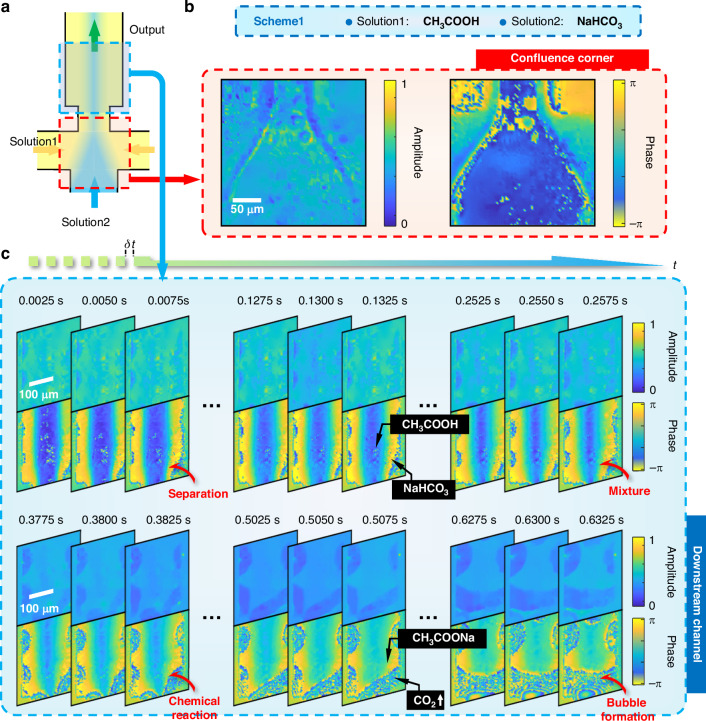
Experimental demonstration of FACE-SPCM in a chemical reaction. **a** Schematic of the microfluidic device designed for mixing different liquids, highlighting the microstructure at the confluence corner (red) and the downstream channels (blue). Scheme 1: Acetic acid (Solution 1) and baking soda (Solution 2) are used to demonstrate a chemical reaction. **b** Complex-field microscopy at the confluence corner. Scale bar: 50 μm. Average CNR: 4.98. **c** Reconstructed complex-field microscopy captured in real-time sequences over short and long temporal intervals. The entire process of the acid-base neutralization reaction is visualized, including separation, mixing, chemical reaction, and CO_2_ bubble formation, with detailed labeling of the chemical components. Scale bar: 100 μm. Average CNR: 4.70

After monitoring the dynamics of microfluidics and live microorganisms, the FACE-SPCM system demonstrated its potential for real-time analysis of chemical reactions in transparent solutions, leveraging robust phase information retrieval and high-speed imaging capabilities. Unlike biological samples containing fats and proteins that show slight absorptive contrast due to NIR specialization, uniform water-based solutions pose an additional challenge in visualizing transparent details. To demonstrate the system’s capability to discern chemical interactions, we designed two experimental schemes: Scheme 1 for a chemical reaction and Scheme 2 for a simple physical mixture. Both schemes involve two transparent solutions, labeled Solution 1 and Solution 2. As shown in Fig. 4a, the microfluidic chip was specifically designed to efficiently mix the two chemical reagents: Solution 1 enters through the side inlets and Solution 2 through the bottom inlet. The confluence corner, where the solutions initially meet, is highlighted in a red box, while the downstream channel, where mixing continues, is marked in a blue box. To ensure stable mixing, an automatic syringe pump injected Solution 1 at 2 mL h^−1^ from the side inlets and Solution 2 at 1.3 mL h^−1^ from the bottom inlet. A detailed microstructure of this microfluidic chip specifying solution mixing is provided in Supplementary Note 11.

As shown in Fig. 4, Scheme 1 involves a chemical reaction between acetic acid (CH_3_COOH) as Solution 1 and baking soda (NaHCO_3_) as Solution 2, resulting in an acid-base neutralization reaction that produces sodium acetate (CH_3_COONa) and carbon dioxide gas (CO_2_). Using high-precision settings, our SPCM system captured detailed amplitude and phase images at the confluence corner (red box in Fig. 4a), as shown in Fig. 4b. Due to the high flow rates at the confluence corner (2 mL h^−1^ for Solution 1 and 1.3 mL h^−1^ for Solution 2), the reaction was not fully completed at this location. Instead, the main site of reaction and mixing occurred further downstream (blue box in Fig. 4a), which was more suitable for observing the dynamics with the SPCM system. A subset of real-time complex-field imaging results is shown in Fig. 4c, with specific elapsed times indicated above each image. Because of the solutions’ transparency, amplitude imaging provides minimal detail, whereas phase imaging reveals significant contrast due to differences in optical refractive indices. Figure 4c shows CH_3_COOH in the central region flanked by NaHCO_3_ on both sides. As these solutions merged and flowed, their distinct phase boundaries gradually blurred, indicating the ongoing chemical reaction that forms CH_3_COONa and CO_2_, as evidenced by bubble formation within the mixture.

**Fig. 5:**
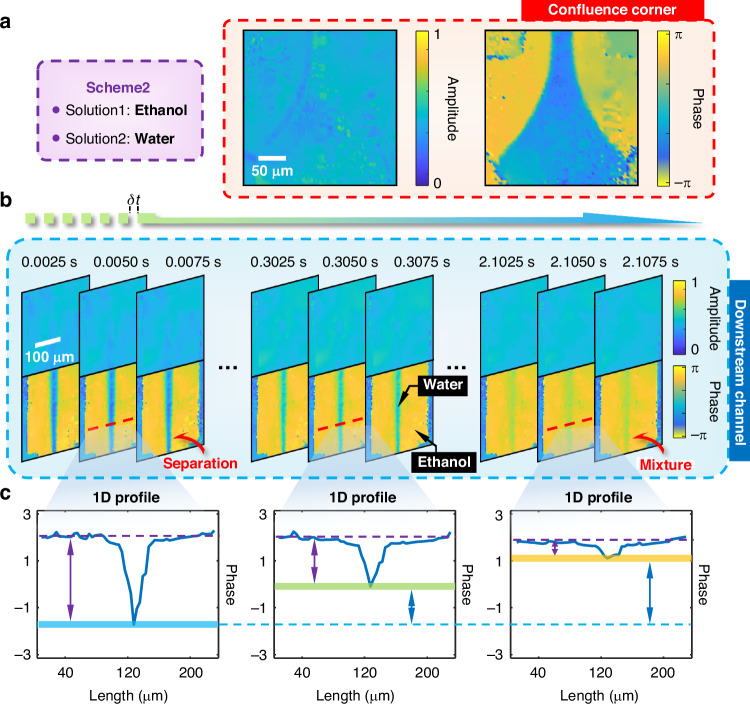
Experimental demonstration of FACE-SPCM in solution mixture. **a** Scheme 2: Ethanol (Solution 1) and pure water (Solution 2) are selected to illustrate a non-reactive chemical scenario. Corresponding complex-field microscopy at the confluence corner is shown. Scale bar: 50 μm. Average CNR: 4.86. **b** Reconstructed complex-field microscopy captured in real-time sequences over both short and long temporal intervals. The entire mixing process of ethanol and water is visualized, depicting the separation and mixing stages without any detectable chemical reactions. The involved ingredients are clearly labeled. Scale bar: 100 μm. Average CNR: 1.17. **c** 1D profiles of phase images across different stages, quantitatively showing the continuous evolution of the ethanol–water mixture

Conversely, as a control group without chemical reactions, Scheme 2 was prepared using ethanol (C_2_H_5_OH) as Solution 1 and pure water (H_2_O) as Solution 2, as shown in Fig. 5. Using the same microfluidic setup, the primary sites for real-time analysis with the SPCM system were at the confluence corner and the downstream channel, highlighted by the same red and blue boxes in Fig. 4a, respectively. At the confluence corner displayed in Fig. 5a, phase contrast reveals a distinct separation between the solutions due to their high flow rates, while complete mixing occurs in the downstream channel. Thus, as illustrated in Fig. 5b, this setup allows for real-time monitoring of the ethanol-water mixing process with complex-field reconstructions at specific elapsed times. Unlike the minimal changes in amplitude, the phase boundaries between C_2_H_5_OH and H_2_O gradually blur as mixing progresses, highlighting the difference between a homogeneous mixture and a chemical reaction. Figure 5c shows the 1D profiles along the red dotted lines from the phase images above, quantitatively demonstrating a gradual decrease in phase contrast as mixing continues, providing additional visual confirmation.

These results demonstrate the effectiveness of the FACE-SPCM system in observing and analyzing real-time chemical reactions and solution mixing over short durations. Additional dynamic visualizations of these chemical processes, slowed down by a factor of eight, are available in Supplementary Movie S5. The detailed preparation of the chemical solutions is described in the “Methods” section.

## Discussion

The FACE-SPCM system demonstrates excellent scalability and adaptability for various dynamic conditions. Its scalability spans different imaging setups and spectral configurations, largely due to the flexibility of the FACE probing structure. For example, Imaging Scheme 2 is optimized for monitoring dynamic structures with a large FoV, whereas Imaging Scheme 3 prioritizes image quality with minimal aberrations in scenarios that do not require ultrafast observation. When scaling the system, inherent trade-offs between imaging parameters must be considered. First, as in many other imaging systems, there is a linear proportional relationship between the value of resolution and FoV. Second, an inverse linear relationship exists between frame size and FPS. For a given acousto-optic modulation bandwidth, increasing the number of pixels requires resolving a correspondingly larger number of frequency components. This, in turn, necessitates a longer measurement time series based on the principles of the Fourier transformation, thereby reducing the achievable FPS. A quantitative analysis of this compromise is provided in Supplementary Note 13. With these two trade-offs in mind, the system design can be strategically scaled to accommodate different imaging requirements. Besides, considering that computational imaging has demonstrated improvements in both resolution and FPS through compressive sensing^54^, we envision that by digitally redesigning the FACE pattern with the accommodation of probing structure, these concepts could also contribute to enhancing the imaging performance of FACE-SPCM in future developments.

While this study demonstrates the system at 1030 nm in the NIR range, the FACE scheme is not restricted to this wavelength. In future implementations, it can be readily adapted to other spectral ranges by modifying the excitation light source, AODs, corresponding optical components, and single-pixel detectors. Currently, commercial AOD technologies support operation across a broad spectral range, from ultraviolet to mid-infrared (380–2100 nm), while suitable light sources, optical elements, and single-pixel detectors are available over an even wider range. Further increasing the frame size while maintaining the reported FPS is currently constrained by the modulation bandwidth of the AODs. If future advancements enable AODs with GHz-level modulation bandwidth while maintaining comparable performance, the frame size could be increased by two to three orders of magnitude. In such a scenario, beat frequencies in the GHz range would need to be measured, which would exceed the capabilities of the current single-pixel detectors operating in the MHz range. To accommodate this, higher-bandwidth single-pixel detectors capable of GHz-range operation—already commercially available—would be required.

The proposed FACE scheme ensures efficiency in both spatial and temporal domains, providing high adaptability across various complex scenarios. For spatial imaging, the FACE pattern enables a wide 2D range to capture global imaging details in an ultrashort time, making it well-suited for dynamic processes beyond 1D scanning-based cytometry applications. For real-time imaging over extended periods, the system supports continuous data acquisition and processing, with storage and computation managed via a buffer at the computing terminal. This setup allows for long-term monitoring, constrained only by memory capacity. For example, storing a one-minute video at a 50 MHz sampling rate requires approximately 3 GB of data.

In summary, the developed FACE-SPCM system demonstrates exceptional real-time monitoring capabilities for complex-field microscopy in the NIR spectrum, achieving an FPS of 1000 Hz, a lateral resolution of 3.76 μm, a FoV of approximately 300 μm, and a record-high SBP-T of 1.3 × 10^7^. A series of experimental demonstrations—including the real-time monitoring of microfluidics, live microorganisms, and chemical reactions—validate the system’s adaptability and effectiveness in capturing phase information in both transparent and scattering environments. The common-path interference structure enhances performance by minimizing environmental disturbances. The FACE scheme significantly boosts the information throughput of systems using single-pixel detectors, enhancing their capacity for real-time complex-field monitoring in the NIR spectrum. This innovative technology offers broad utility across diverse scientific and industrial applications, underscoring its transformative impact and versatility.

## Materials and methods

### Imaging reconstruction

The illumination framework of the FACE scheme involves a spectral-encoded projection ${E}_{{\rm{S}}}$ with specified frequency tones $f\left({x}_{n},{y}_{m}\right)$ and an unmodulated component ${E}_{{\rm{R}}}$ with a base frequency ${f}_{0}$. When this illumination interacts with the complex-field object $O\left({x}_{n},{y}_{m}\right)$, the resulting signal, carrying the encoded information, is captured by the single-pixel photodetector. The interaction between the different frequency tones and the unmodulated base frequency creates field interference, resulting in a complex heterodyne holography signal. This signal can be mathematically represented in a simplified form, as shown in Eq. 3, through sideband intensity detection. However, in practical scenarios, there are residual 1D diffractions ${E}_{1{\rm{D}}}$ with frequencies ${f}_{x}\left({x}_{n}\right)$ due to the inherent architecture. As discussed in detail in Supplementary Note 3, the polarization effects are considered, and a comprehensive form can be derived for the full characterization of the cosine term as follows:

4 $$\begin{array}{lll}I\left(t\right)={\left|{E}_{{\rm{R}}}\right|}^{2}+\mathop{\sum }\limits_{n,m}{\left|{E}_{{\rm{S}}}O\left({x}_{n},{y}_{m}\right)\right|}^{2}+\mathop{\sum }\limits_{n}{\left|{E}_{1{\rm{D}}}\right|}^{2}\\\qquad+\,2{E}_{{\rm{S}}}{E}_{{\rm{R}}}\mathop{\sum }\limits_{n,m}O\left({x}_{n},{y}_{m}\right)\cos \left(2\pi \left(f\left({x}_{n},{y}_{m}\right)-{f}_{0}\right)t\right)\\\qquad+\,{2\left|{E}_{{\rm{S}}}\right|}^{2}\mathop{\sum }\limits_{n,m}\mathop{\sum }\limits_{n^{\prime} ,m^{\prime} \in \left(n^{\prime} ,m^{\prime} \right)\ne \left(n,m\right)}{\mathrm{Re}}\left(O\left({x}_{n},{y}_{m}\right){O}^{* }\left({x}_{n^{\prime} },{y}_{m^{\prime} }\right)\right)\\\qquad\quad\,\cos \left(2\pi \left(f\left({x}_{n},{y}_{m}\right)-f\left({x}_{n^{\prime} },{y}_{m^{\prime} }\right)\right)t\right)\\\qquad+\,2{\left|{E}_{1{\rm{D}}}\right|}^{2}\mathop{\sum }\limits_{n}\mathop{\sum }\limits_{n^{\prime} \in n^{\prime} \ne n}\cos \left(2\pi \left({f}_{x}\left({x}_{n}\right)-{f}_{x}\left({x}_{n^{\prime} }\right)\right)t\right)\end{array}$$

We then process the signal using FFT, expressed as $E\left(f\right)=\int I\left(t\right)\exp \left(-i2\pi {ft}\right){dt}$. Given the definition $\cos \left({ft}\right)=(\exp \left({ift}\right)+\exp \left(-{ift}\right))/2$ and the Dirac delta function $\delta \left(f-f^{\prime} \right)=\int \exp \left({if}^{\prime} t\right)\exp \left(-{ift}\right){dt}$, an updated representation in the frequency domain is developed as:

5 $$\begin{array}{l}E\left(f\right)={\rm{DC}}\delta \left({f}\right)+{E}_{{\rm{S}}}{E}_{{\rm{R}}}\mathop{\sum }\limits_{n,m}O\left({x}_{n},{y}_{m}\right)\left(\delta \left(f-\left(f\left({x}_{n},{y}_{m}\right)-{f}_{0}\right)\right)\right.\\\qquad\;\;+\left.\delta \left(f+\left(f\left({x}_{n},{y}_{m}\right)-{f}_{0}\right)\right)\right)+{2\left|{E}_{{\rm{S}}}\right|}^{2}\mathop{\sum }\limits_{n,m}\mathop{\sum }\limits_{n^{\prime} ,m^{\prime} \in \left(n^{\prime} ,m^{\prime} \right)\ne \left(n,m\right)}\\\qquad\quad\;{\mathrm{Re}}(O\left({x}_{n},{y}_{m}\right){O}^{* }\left({x}_{{n}^{{\prime} }},{y}_{{m}^{{\prime} }}\right))\left(\delta \left(f-\left(f\left({x}_{n},{y}_{m}\right)-f\left({x}_{{n}^{{\prime} }},{y}_{{m}^{{\prime} }}\right)\right)\right)\right.\\\qquad\;\;+\left.\delta (f+\left(f\left({x}_{n},{y}_{m}\right)-f\left({x}_{n^{\prime} },{y}_{m^{\prime} }\right)\right))\right)\\\qquad\;\;+2{\left|{E}_{1{\rm{D}}}\right|}^{2}\mathop{\sum }\limits_{n}\mathop{\sum }\limits_{n^{\prime} \in n^{\prime} \ne n}\left(\delta (f-\left({f}_{x}\left({x}_{n}\right)-{f}_{x}\left({x}_{{n}^{{\prime} }}\right)\right))\right.\\\qquad\;\;+\left.\delta (f+\left({f}_{x}\left({x}_{n}\right)-{f}_{x}\left({x}_{n{\prime} }\right)\right))\right)\end{array}$$

where DC represents the synthesis of all direct current terms during the beat frequency of heterodyne holography. The cleavage of frequency components, such as the twin terms $\delta ({f}\mp \left({f}\left({x}_{n},{y}_{m}\right)-{f}_{0}\right))$, highlights the essence of side-band detection resulting from the cosine representation. As detailed in Supplementary Note 4, thanks to the asymmetry in the number of tones along orthogonal directions, varied frequency spacing and subtle frequency shifts jointly ensure a rigorous one-to-one spatial-frequency mapping, preventing spectral aliasing during the coherent detection. This is particularly evident in the inequality $\delta (f-\left(f\left({x}_{n},{y}_{m}\right)-{f}_{0}\right))\ne \delta (f+\left(f\left({x}_{n{\prime} },{y}_{m{\prime} }\right)-{f}_{0}\right)$. As derived from Eq. 5, the complex-field information from the object is directly encoded in the second term. By leveraging the known beat frequency $f\left({x}_{n},{y}_{m}\right)-{f}_{0}$, the complex-field object can be retrieved as:

6 $$E\left(f\left({x}_{n},{y}_{m}\right)-{f}_{0}\right)={E}_{{\rm{S}}}{E}_{{\rm{R}}}O\left({x}_{n},{y}_{m}\right)\propto O\left({x}_{n},{y}_{m}\right)$$

### Time consumption in complex-field imaging reconstruction

Real-time monitoring with the FACE-SPCM system involves two key components: data acquisition and data processing, both executed in a streamlined manner. For a well-optimized configuration of the FACE scheme (e.g., Imaging Scheme 1), the data acquisition time for a single reconstruction is 1 ms. Data processing is performed in parallel with data acquisition using a central processing unit (CPU: Intel i7-13700K) and a high-speed graphics processing unit (GPU: NVIDIA RTX 4070Ti). The time required for the FFT operation is approximately 282 μs, the calibration operation takes around 36 μs, and the index extraction operation for display is about 13 μs. Consequently, the FACE-SPCM system enables real-time imaging at 1000 Hz during streamlined data acquisition and processing. The detailed workflow is provided in Supplementary Note 14.

### Experimental setup

The experimental configuration of the FACE-SPCM system is schematically illustrated in Fig. 2a. A continuous-wave laser (DHNL 1030.10-50-P-N-M-FA) with an ultra-narrow bandwidth (~3 kHz at 1030 nm) served as the stable probing source up to 400 mW. A quarter-wave plate and a half-wave plate ensured proper linear polarization alignment, while a polarization beam splitter regulated the laser intensity. The laser light was directed via mirrors and expanded using lenses with focal lengths of 8 mm and 60 mm before reaching the acousto-optic deflectors (AODs) (DTSX-400-1064, AA Opto-Electronic). An iris with a 5 mm diameter filtered the expanded beam to fit the entrance of the AODs, optimizing the time-bandwidth product through two-dimensional (2D) Bragg diffraction in the FACE scheme. The time-bandwidth product, $N=D/V\Delta F$, related to the effective number of modulation branches and was proportional to the input beam diameter under efficient modulation. The AODs were driven by a tailored frequency-comb electronic signal from a function generator (DG4102, RIGOL), which was amplified by amplifiers (AMPB-B-30-10.500, AA Opto-Electronic). The frequency comb for AODx spanned 80 tones (63–88 MHz), while AODy covered 81 tones (63–88 MHz) within the allowed 61–91 MHz bandwidth. The encoding strategy, inspired by orthogonal frequency division multiplexing (OFDM) principles, is detailed in Supplementary Note 4. After mirror adjustments, the expanded beam underwent 2D Bragg diffraction via the AODs at an optimal angle (~120 mrad). AODx generated diffracted branches, which were further expanded into a 2D sub-beam array by AODy, while maintaining an unmodulated component for common-path interference. A 4*f* system, composed of identical lenses with a 125 mm focal length, ensured precise pattern alignment and convergence, as detailed in Supplementary Note 2. The resulting 2D sub-beam array scanned angles from 0 to 140 mrad in both the x and y directions, achieving spectral-encoded projection with distinct temporal oscillations. The relationship between frequency and angle was central to the innovative aspect of the FACE scheme. After passing through the AODs, the beam was directed through an imaging lens (7.5 mm focal length) and an acquisition lens (25 mm focal length), forming another 4*f* system. The imaging lens collected all beams at the confocal plane to interact with the sample for complex-field microscopy, while the acquisition lens reconverged the interacted beams at the rear focal plane of the 4*f* system. During imaging, the 2D sub-beam array had a relatively low power of approximately 100 μW and was projected over an area of 300 μm, resulting in an optical intensity of 110 mW cm^−2^, which is below the American National Standards Institute safety limit of 1000 mW cm^−2^ for maximum permissible exposure at near-infrared wavelengths. A 50 lp mm^−1^ grating was used for unified direction in common-path heterodyne holography, as described in Supplementary Note 6. The combined beam was then directed to a single-pixel photodetector (APD130C, Thorlabs) for signal collection. Real-time data acquisition was performed using a data acquisition card (ATS9462, AlazarTech), configured with a sampling rate of 50 MSa s^−1^.

### Experimental preparation for living microorganisms and chemical reactions

The biological demonstration focuses on real-time monitoring of the living microorganism Paramecium. Paramecia were cultured in a nutrient-rich medium under well-lit conditions at temperatures of 20–25 °C and harvested after 2–3 days of growth. A small drop of the culture was collected with a pipette and placed on a clean glass slide. To maintain the paramecia’s vitality for extended observation, a coverslip was not applied over the liquid drop. For the chemical analysis, two water-based solution mixtures were monitored to determine the occurrence of chemical reactions, organized into two schemes. Scheme 1 examines the reaction between acetic acid (CH_3_COOH) and sodium bicarbonate (NaHCO_3_). Both reactants were prepared at equal concentrations to ensure effective acid-base neutralization. A 100 mL solution of 5% NaHCO₃ was prepared by dissolving 5 g of pure sodium bicarbonate powder in water and filling to the 100 mL mark in a graduated cylinder. Similarly, a 100 mL solution of 5% acetic acid was prepared by diluting a 10% acetic acid solution to the required concentration. Scheme 2 serves as a control, using a mixture of ethanol (C_2_H_5_OH) and water (H_2_O), where no chemical reaction occurs. For this scheme, 100 mL of absolute ethanol was prepared without water, and pure water was used as the H_2_O component.

## Supplementary information

Supplementary Information for “Ultrahigh-throughput single-pixel complex-field microscopy with frequency-comb acousto-optic coherent encoding (FACE)”

Supplementary Movie S1 bright-field microscopy

Supplementary Movie S2 bubble

Supplementary Movie S2 droplet

Supplementary Movie S3 droplet_scat

Supplementary Movie S4 Paramecium

Supplementary Movie S5 acid-neutralization reaction

Supplementary Movie S5 ethanol-water mixture

## Data Availability

The relevant raw data and codes that produce all relative video and imaging results supporting this work are publicly available on public repository Zenodo: 10.5281/zenodo.14087848.
