# Supplementary material for: Ultrahigh-throughput single-pixel complex-field microscopy with frequency-comb acousto-optic coherent encoding (FACE)
Source: Light Sci Appl. 2025 Aug 11;14:266. doi: 10.1038/s41377-025-01931-w (PMC12340123; doi:10.1038/s41377-025-01931-w)
Supplement: Supplementary file 1 — Supplementary Information for “Ultrahigh-throughput single-pixel complex-field microscopy with frequency-comb acousto-optic coherent encoding (FACE)” [file 41377_2025_1931_MOESM1_ESM.docx]

**Supplementary Information for “Ultrahigh-throughput single-pixel complex-field microscopy with frequency-comb acousto-optic coherent encoding (FACE)”**

Daixuan Wu^1^, Yuecheng Shen^2,*^, Zhongzheng Zhu^1^, Tijian Li^1^, Jiawei Luo^2^, Zhengyang Wang^2,3^, Jiaming Liang^2,3^, Zhiling Zhang^2^, Yunhua Yao^2^, Dalong Qi^2^, Lianzhong Deng^2^, Zhenrong Sun^2^, Meng Liu^1^, Zhi-Chao Luo^1,4,*^, and Shian Zhang^2,5,6,*^

^1^*Guangdong Provincial Key Laboratory of Nanophotonic Functional Materials and Devices, Guangdong Basic Research Center of Excellence for Structure and Fundamental Interactions of Matter, School of Optoelectronic Science and Engineering, South China Normal University, Guangzhou, Guangdong 510006, China.*

^2^*State Key Laboratory of Precision Spectroscopy, School of Physics and Electronic Science, East China Normal University, Shanghai 200241, China.*

^3^*School of Electronics and Information Technology, Sun Yat-sen University, Guangzhou 510006, China.*

^4^*MOE Key Laboratory of Laser Life Science, School of Optoelectronic Science and Engineering, South China Normal University, Guangzhou 510631, China.*

^5^*Joint Research Center of Light Manipulation Science and Photonic Integrated Chip of East China Normal University and Shandong Normal University, East China Normal University, Shanghai 200241, China.*

^6^*Collaborative Innovation Center of Extreme Optics, Shanxi University, Taiyuan 030006, China.*

Corresponding authors: [ycshen@lps.ecnu.edu.cn](mailto:ycshen@lps.ecnu.edu.cn); [zcluo@scnu.edu.cn](mailto:zcluo@scnu.edu.cn); sazhang@phy.ecnu.edu.cn;

**Supplementary Note 1: Comparative analysis of high-throughput real-time streamlined imaging systems**

To prove the real-time streamlined superiority, a comprehensive comparison of the developed frequency-comb acousto-optic coherent encoding (FACE) single-pixel complex-field microscopy (SPCM) system throughout the existing high-throughput real-time single-pixel imaging (SPI) and SPCM systems is organized in Supplementary Table 1. This table includes key imaging parameters such as frame size, frames per second (FPS), imaging resolution, operational spectrum, and imaging type. Additionally, a comparison with a commercial near-infrared (NIR) camera (008TNIR) is provided to highlight potential applications in the NIR spectrum.

To quantify the throughput of the imaging system, we introduce the space-bandwidth-time product (SBP-T) as a figure of merit, characterizing the imaging information collected by the streamlined systems per unit time. This metric primarily depends on two key parameters: frame size and FPS. The FPS indicates the imaging speed, while the frame size determines the amount of information in each imaging frame. Mathematically, the SBP-T is calculated by multiplying the FPS (excluding the effects of compressive sensing) by the frame size for amplitude-only images. For complex-field images, the SBP-T is doubled to account for the inclusion of the phase component.

**Supplementary Tab. 1 Representative high-throughput real-time streamlined imaging systems in the literature**

| **Reference** | **Imaging type** | **SBP-T** | **FPS** | **Frame size** | **Resolution** | **Spectrum** |
| --- | --- | --- | --- | --- | --- | --- |
| Ref. [1] | Amplitude | 7.5×10^5^ | 72 Hz | 157 × 159 | 2.6 μm | Visible |
| Ref. [2] | Amplitude | 1.8×10^5^ | 51 Hz | 59 × 61 | 88.4 μm | Visible |
| Ref. [3] | Complex field | 4.6×10^4^ | 0.35 Hz | 256 × 256 | 4.3 μm | Visible |
| **FACE-SPCM (This work)** | Complex field | **1.3×10^7^** | **1,000 Hz** | 80 × 81 | 3.8 μm | NIR |
| InGaAs Camera (008TNIR) | Amplitude | 7.4×10^6^ | 90 Hz | 320 × 256 | 30 μm | NIR |

SBP-T = FPS × Frame size × Imaging type (Amplitude: 1, Complex field: 2)

Although the systems reported in Refs. [1, 2] increased the SBP-T to approximately ~10⁵ by employing new projection schemes that overcome the limitations of digital micromirror devices (DMDs), they are limited to forming amplitude-only images. The highest SBP-T achieved for complex-field imaging to date was through heterodyne holography, which raised the DMD-based SBP-T to 4.6 × 10⁴ [3]. However, this method is still constrained by the inherent limitations of the DMD structure, resulting in significantly limited imaging speed. Furthermore, despite the benefits of single-pixel detection schemes, no high-throughput, real-time SPI/SPCM systems have been demonstrated for imaging beyond the visible spectrum. By employing the FACE scheme, the developed FACE-SPCM system surpasses the information throughput of previous SPCM (~10⁴), SPI (~10⁵), and even commercial cameras (~10⁶) operating in the NIR spectrum. Notably, in addition to achieving record-high throughput, FACE-SPCM enables ultrahigh-throughput streaming at 1,000 FPS, with an SBP-T of up to ~10⁷.

Moreover, considering over generality of single-pixel detection, time-stretch (TS) techniques with spectral shower projection are under our reasonable discussion [4-6], demonstrating ultrahigh-throughput imaging capabilities. However, this approach requires costly ultrafast light sources and follows a fundamentally different technological pathway from FACE-SPCM (operates with a single-frequency laser), resulting in rigid grating designs with finite frame size. Alternatively, fluorescence imaging using radiofrequency-tagged emission (FIRE) techniques shares a similar conceptual basis but is peculiar to one-dimensional (1D) imaging, specifying the real-time cell sorting through the scanning-based flow cytometry [7, 8]. Given these high-throughput regimes for intensity signal recording, an additional comparison is provided in Supplementary Table 2 for a broader discussion.

**Supplementary Tab. 2 Additional comparison of TS techniques and FIRE techniques. All these systems have been demonstrated exclusively for amplitude-only imaging.**

|  | **Key feature** | **Reference** | **SBP-T** | **FPS** | **Frame size** | **Resolution** | **Spectrum** |
| --- | --- | --- | --- | --- | --- | --- | --- |
| TS techniques | Ultrafast light source | Ref. [4] | 1.4×10^10^ | 6.1 MHz | 90 × 25 | 10 μm | NIR |
|  |  | Ref. [5] | 1.2×10^9^ | 10 MHz | 1 × 120 | 0.8 μm | Visible |
|  |  | Ref. [6] | 2.0×10^7^ | 500 kHz | 1 × 40 | 0.5 mm | NIR |
| FIRE techniques | One-dimensional image | Ref. [7] | 1.0×10^8^ | 800 kHz | 1 × 125 | 5.9 μm | Visible |
|  |  | Ref. [8] | 1.6×10^6^ | 15 kHz | 1 × 104 | 1.5 μm | Visible |

SBP-T = FPS × Frame size

**Supplementary Note 2: Acousto-optic Bragg diffraction: physical principles and illumination geometry**

This section describes the optical setup of the FACE scheme, which is implemented using a relay system with a pair of orthogonal acousto-optic deflectors (AODs). The acousto-optic crystal, optimized for the NIR spectrum in this study, features a narrow, bar-shaped entrance for input illumination to ensure high efficiency in frequency-shifting modulation. In an acousto-optic crystal, two major types of diffraction can occur: Raman-Nath diffraction and Bragg diffraction, each characterized by specific conditions. Raman-Nath diffraction occurs when the crystal behaves like a thin grating, resulting in multiple diffracted orders with low intensity in the main order. In contrast, Bragg diffraction takes place in a volume grating, where the first-order diffracted beam is maximized through constructive interference with a defined period.

The figure-of-merit parameter $Q$ is introduced to distinguish between Raman-Nath and Bragg diffraction modes, defined as [9]:

$\begin{matrix} Q={2\pi\lambda LF^{2}}/{nV^{2}}={2\pi\lambda L}/{n\Lambda^{2}} \\ \mathrm{where}\left\{ \begin{matrix} Q\ll{2\pi}/n for Raman-Nath mode \\ Q\gg{2\pi}/n for Bragg mode \end{matrix} \right. \end{matrix}$ (S1)

Here, $L$ represents the length of the crystal along the input propagation direction, $\lambda$ is the light wavelength of the input beam, and $\Lambda$ is the sound wavelength in the crystal determined by the driving radiofrequency $F$ and acoustic velocity $V$, analogous to the line spacing of an acoustically driven grating. The parameter $L$ defines the grating-like structures that determine the diffraction type, establishing a criterion of $L\gg{\Lambda^{2}}/\lambda$ (or $L\ll{\Lambda^{2}}/\lambda$). This criterion reflects the narrow bar shape of the acousto-optic crystal, and in this work, the diffraction falls within the Bragg diffraction category.

In 1D Bragg diffraction, a series of frequency-shifting diffractions are driven by a specified frequency comb, causing sub-beam illumination with different frequencies to deflect from the original direction. The relationship between the frequency shift $\Delta F$ and the deflecting angle $\Delta\theta$ is given by:

$\Delta\theta=\lambda{\Delta F}/V$ (S2)

By adjusting the interaction angle between the acousto-optic crystal and the input illumination, the ±1 Bragg orders can be switched, leading to a down-conversion in frequency.


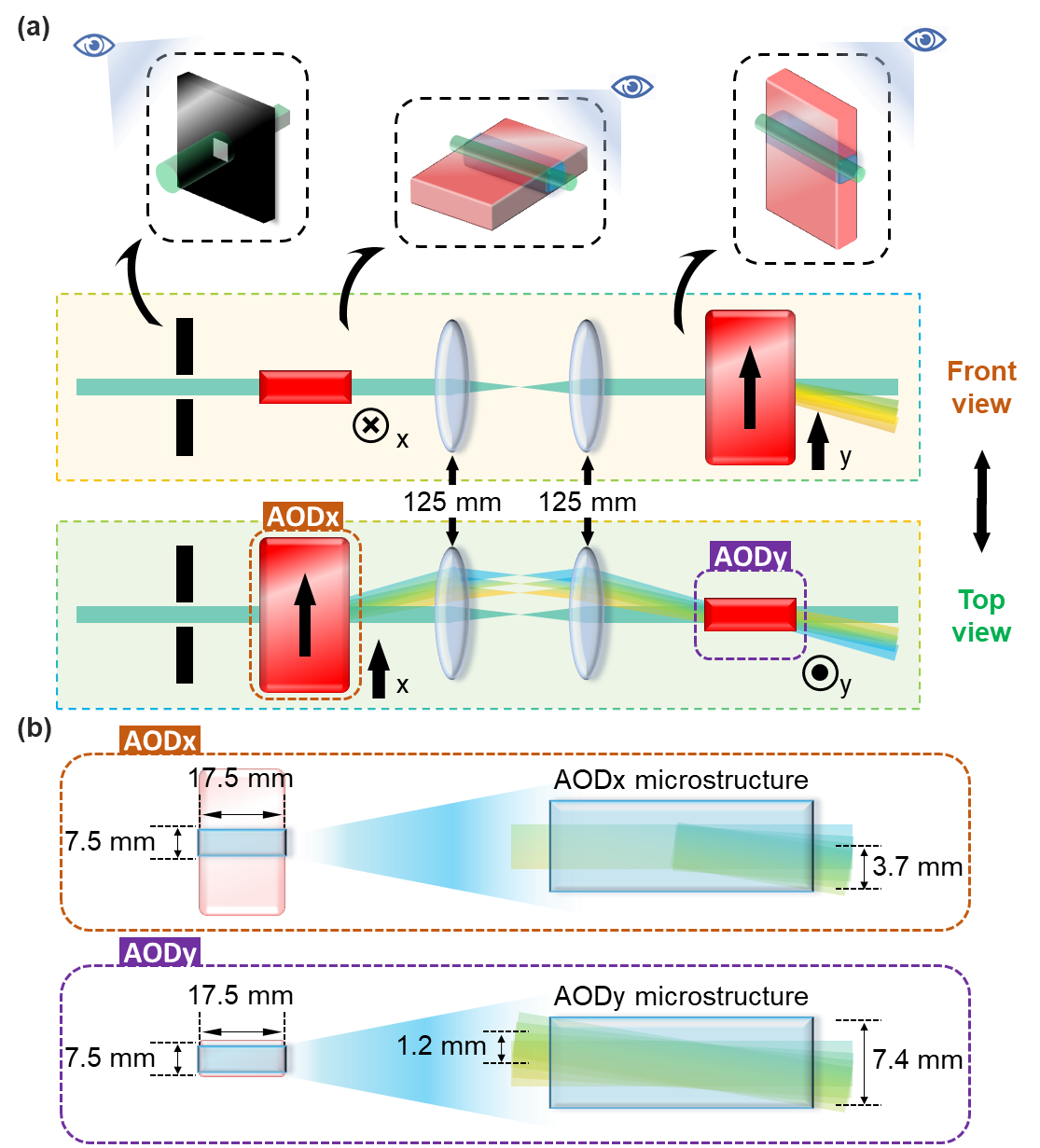


**Figure S1 | Illumination framework for acousto-optic Bragg diffraction.** (a) The experimental setup includes a finely tuned 4*f* system that relays the propagating Bragg diffraction between a pair of orthogonal AODs, as illustrated in the front and top views. (b) The microstructure of the twin AODs is designed to generate spectral-encoded projections while maintaining structural tolerance to preserve the unmodulated zero-order beam.

For practical implementation, we used commercial AODs (DTSX-400-1064, AA Opto-Electronic) with a high center modulating frequency of 76 MHz, optimized for 1030 nm, achieving a deflection angle of approximately 120 mrad. This angle is crucial for a detailed examination of the microstructure. As shown in Fig. S1a, the front and top views provide a comprehensive perspective of how the illumination enters the crystal and generates a series of side branches driven by the frequency comb. The top view shows that the expanded branches from the first AOD in the x direction are redirected into the second AOD via a carefully designed 4*f* system, avoiding any interference. The front view illustrates the further expansion of acousto-optic branches in the y direction, forming a complete two-dimensional (2D) spectral-encoded projection. However, the FACE-SPCM system requires preserving the zero-order beam for subsequent coaxial heterodyne holography synthesis. Therefore, both the main path and the generated branches of the illumination must be reshaped to align with the configuration of the twin AODs, imposing stricter design requirements on the propagation geometry.

To ensure successful passage through the twin AODs, Fig. S1b details their microstructures. For the first AOD operating in the x direction (AODx), a limited lateral aperture is created using an iris to shape the illumination into a 5-mm side-length square, matching the lateral entrance of the AODs for precise modulation. Given the crystal length of approximately 17.5 mm, deflection in the middle of AODy results in a lateral displacement of about 1.2 mm on one side, occupying around 3.7 mm, which is less than half the crystal width of approximately 3.75 mm. Without correction, the diverging branches of Bragg beams exiting AODx would miss the subsequent AOD operating in the y direction (AODy). To address this, a carefully designed 4*f* system is employed to achieve image conjugation and alignment, accurately forming the FACE pattern. The optical lenses in the 4*f* system have a focal length of 125 mm each. As the zero-order beam and its branches enter the 4*f* system, they become parallel along the main axis, forming a 1D expanding focus array at the confocal plane. The subsequent lens reconverges these parallel sub-beams into AODy at the middle of the crystal, redirecting each divergent wave into a straight Gaussian beam for the next 1D Bragg diffraction. Imaging lenses in the 4*f* system can be substituted with cylindrical lenses positioned along the x direction. With AODy oriented orthogonally at a 90° tilt, the previous 1D expansion allows propagation within a size of approximately 7.4 mm, which is smaller than the crystal height of around 7.5 mm. Each sub-Gaussian beam undergoes Bragg diffraction, generating new branches along the orthogonal dimension and forming a 2D expanding sub-beam array diverging from AODy while maintaining the zero-order beam. By adjusting the spacing between AODy and the lens, the deflection angles in the y direction can be aligned to form a well-defined square shape for imaging. When the emitted sub-beam array is collected by an imaging lens, the divergent illumination is redirected into a 2D parallel focus array at the focal plane, similar to the 1D focus array in a 4*f* system. This setup generates the spectral-encoded projection, which includes the FACE pattern along with the unmodulated zero-order reference required for heterodyne holography.

**Supplementary Note 3: Polarization states of the spectral-encoded projection**

When discussing heterodyne holography in interaction with the established spectral-encoded projection, polarization plays a crucial role in the efficacy of imaging acquisition and data processing. The optical coherence in heterodyne holography during data acquisition depends on the polarized characteristics of the crystals. These crystals, designed for high-resolution deflection, exhibit optical anisotropy in Bragg diffraction, with varying efficiencies across the 61-91 MHz frequency comb depending on the polarization direction. Furthermore, the polarization of the input illumination experiences an orthogonal flip along each axis after Bragg diffraction. Given the complex orthogonal configuration of the twin AODs in the x- and y-directions, a comprehensive visualization from top, front, and side views is provided in Fig. S2 to illustrate these polarization changes.


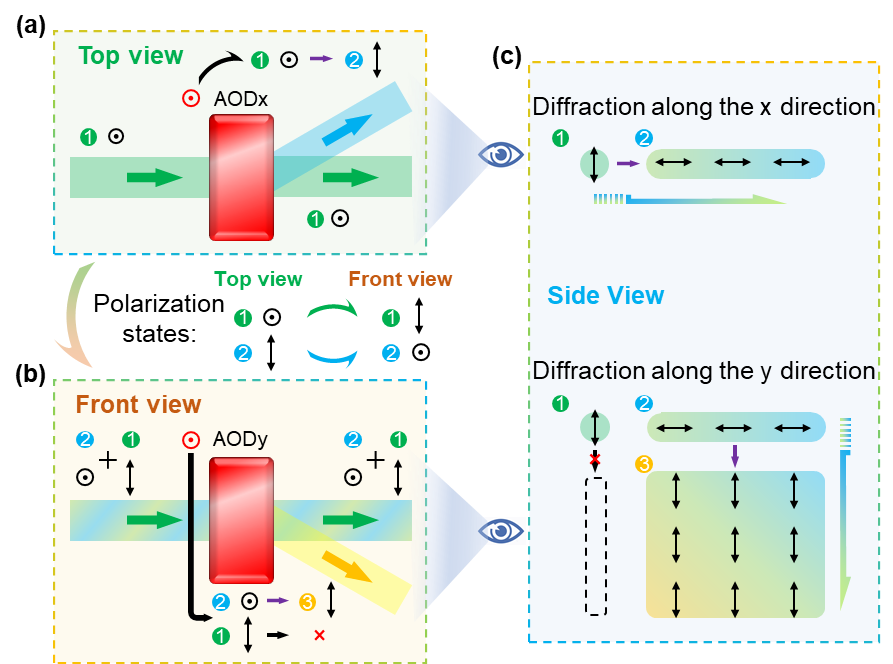


**Figure S2 | Polarization states in the FACE illumination scheme.** (a) The polarization state as light passes through AODx, shown in the top view. (b) The polarization state as light passes through AODy, depicted in the front view, with a visual representation of the polarization direction change from the top view to the front view. (c) The polarization state in the final spectral-encoded projection is displayed in the side view. Polarization state 1 represents the original illumination, polarization state 2 corresponds to the 1D Bragg diffraction branches generated by AODx, and polarization state 3, with double flips, represents the 2D FACE pattern. A postscript explains the visual adaptation of polarization states 1 and 2 switched from the top and front perspectives.

As detailed in Fig. S2a, when the input illumination with appropriate polarization (polarization state 1, marked in green) propagates through AODx, the generated branches undergo a 90° polarization flip (polarization state 2, marked in blue), while the polarization of the unmodulated component remains unchanged (still in polarization state 1). Figure S2 includes a postscript explaining the visual adaptation of polarization states 1 and 2 as viewed from the top and front perspectives. In the front view of Fig. S2b, the orthogonal configuration of AODy allows a similar 90° polarization flip, optimizing modulation efficiency. When the series of modulated branches in polarization state 2 and the unmodulated component in polarization state 1 (which visually overlap in the front view) interact with AODy, the modulated branches are further modulated, generating a 2D FACE pattern that experiences another 90° polarization flip into polarization state 3. The unmodulated component remains unaffected by AODy due to the mismatched polarization direction. As shown in Fig. S2c from the side view, the 2D FACE pattern, having undergone a double polarization flip, aligns with the original unmodulated zero-order beam, maintaining high coherence for effective heterodyne holography. Any residual 1D sub-beams with only a single polarization flip are removed due to incoherence, thereby preventing unintended temporal interference.

**Supplementary Note 4: Orthogonal frequency-comb encoding for the coaxial FACE scheme**

This section discusses the construction of the information bridge between the spatial and frequency domains in the high-speed FACE scheme. The successful demonstration of ultrahigh-throughput FACE-SPCM showcases a unique solution enabled by subsequent heterodyne holography without introducing detrimental ambiguities. This approach relies on a one-to-one mapping between the modulating frequencies generated by 2D Bragg operations and the spatial positions of the probing illuminations, which is fundamental to the FACE principle.

At the core of the FACE scheme is the piezoelectric effect in AODs, driven by a digital-to-analog converter, such as a function generator (FG). The primary challenge is designing the temporal waveform for the two orthogonal radiofrequency signals. Drawing inspiration from orthogonal frequency division multiplexing (OFDM) in optical communications [10], a modified approach is employed to efficiently generate frequency combs with identical spacing, thereby reducing the complexity of the required temporal waveforms. This strategy enhances imaging throughput by using a low-configuration FG to stretch a large number of preloaded frequency teeth. The frequency combs for each orthogonal direction are defined as follows:

$\left\{ f_{x/y} \right\}=f_{\mathrm{int}}:\Delta f:f_{\mathrm{end}}$ (S3)

where $f_{\mathrm{int}}$ and $f_{\mathrm{end}}$ are initially identical for both the x and y directions. The same $\Delta f$ shared in both the x and y direction also indicates the same frequency tones $N=M$.


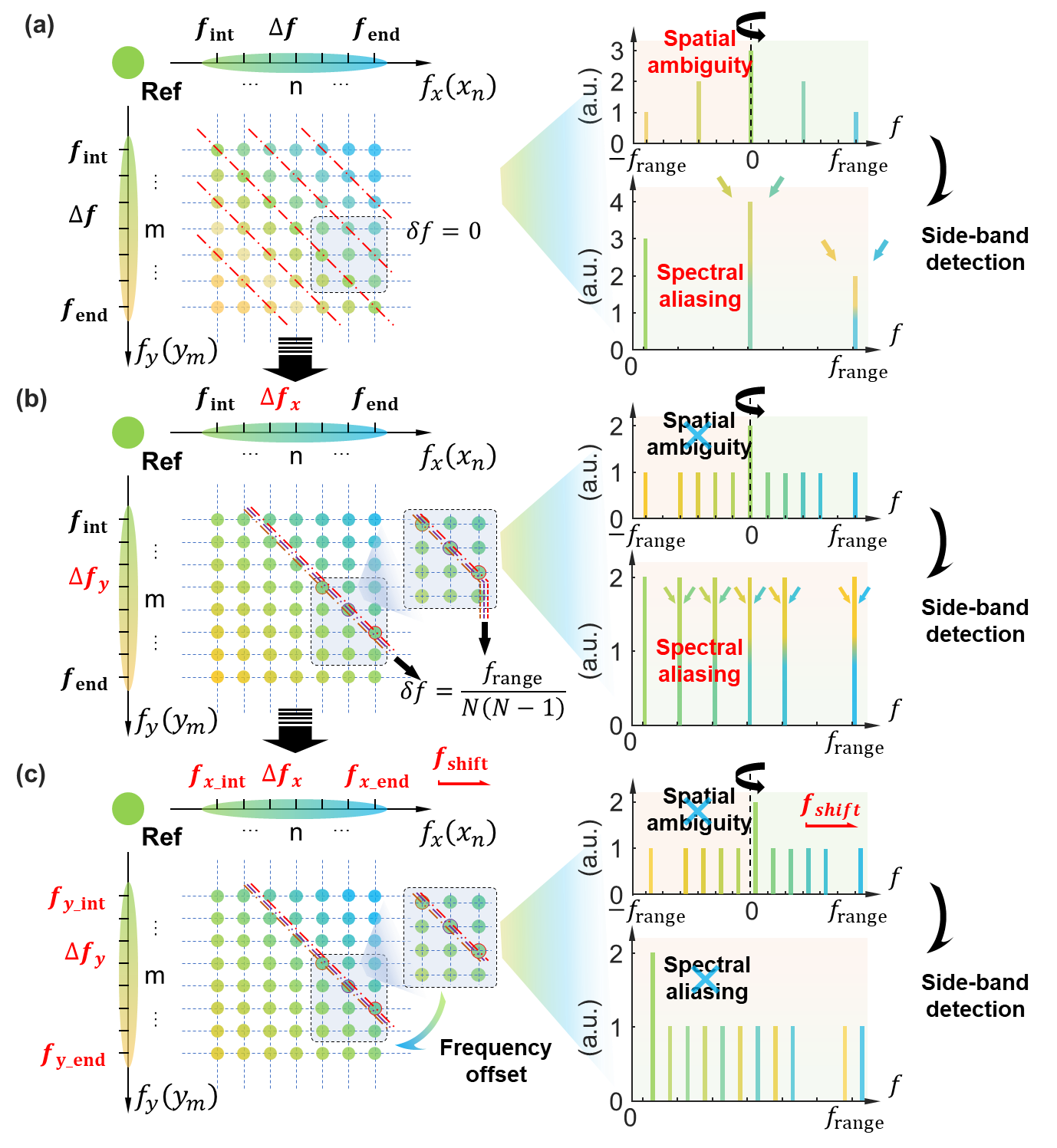


**Figure S3 | Visual illustration of the FACE scheme.** (a) The direct FACE scheme for generating spectral encoding, which suffers from significant issues of spatial ambiguity and spectral aliasing. (b) The orthogonal FACE scheme with misplaced sampling is adopted to eliminate spatial ambiguity and establish a one-to-one mapping; however, it still experiences spectral aliasing due to side-band detection. (c) The utilized FACE scheme, which improves upon previous methods by introducing a subtle frequency offset shift, breaks the bilateral symmetry and effectively avoids spectral aliasing.

In 1D Bragg diffraction, the sub-beam array produced by a single Bragg operation maps the modulating frequency to the ejection angle. In contrast, 2D Bragg diffraction involves double acousto-optic interactions, where each branch of the 2D sub-beam array originates from the corresponding 1D branch of the first diffraction, serving as the new “zero order.” As visualized in Fig. S3a, double acousto-optic interactions introduce a joint frequency shift, expressed as $f=f_{0}+f_{x}+f_{y}$, where $f_{x}$ and $f_{y}$ are elements within $\left\{ f_{x/y} \right\}$ and $f_{0}$ is the base frequency carried by the zero-order beam. This expanded representation of the modulating frequency $f$ corresponds to each diffracted branch at positions $x_{n}$ and $y_{m}$:

$f\left( x_{n},y_{m} \right)=f_{0}+f_{x}\left( x_{n} \right)+f_{y}\left( y_{m} \right)=f_{0}+2f_{\mathrm{int}}+\left( n+m \right)\Delta f$ (S4)

In a practical scenario, to eliminate the term $2f_{\mathrm{int}}$ and achieve successful interference with the zero-order beam, an advanced approach involves reversing one of the AODs. This can be represented as:

$f\left( x_{n},y_{m} \right)=f_{0}+f_{x}\left( x_{n} \right)-f_{y}\left( y_{m} \right)=f_{0}+\left( f_{\mathrm{int}}-f_{\mathrm{int}} \right)+\left( n-m \right)\Delta f$ (S5)

Reversing the modulation order of one of the AODs enables the use of coaxial heterodyne holography in this study, contrasting significantly with similar frameworks that require an additional external local oscillator for reference [11]. However, this modulation scheme can create spatial ambiguities when the frequency shift terms $n+m$ or $n-m$ result in the same value. To address this issue, it is crucial to prevent the overlap of the second term $\left( n-m \right)\Delta f$. This can be achieved by redesigning the frequency intervals $\Delta f_{x}$ and $\Delta f_{y}$ in the x and y directions, respectively. An improved solution involves modifying the number of teeth in the frequency combs to $N$ tones for the x direction and $M=N+1$ tones for the y direction, resulting in different frequency intervals: $\Delta f_{x}={f_{\mathrm{range}}}/\left( N-1 \right)$ and $\Delta f_{y}={f_{\mathrm{range}}}/\left( M-1 \right)={f_{\mathrm{range}}}/N$.

Taking an alternative specification of the frequency combs $\left\{ f_{x}\left( x_{n} \right) \right\}$ and $\left\{ f_{y}\left( y_{m} \right) \right\}$ along orthogonal directions, the modulating frequency in the FACE probing pattern is modified as follows:

$\begin{matrix} f\left( x_{n},y_{m} \right)=f_{0}+n\Delta f_{x}-m\Delta f_{y}=f_{0}+n{f_{\mathrm{range}}}/\left( N-1 \right)-m{f_{\mathrm{range}}}/N \\ \Longrightarrow f_{0}+{f_{\mathrm{range}}}/{N\left( N-1 \right)}\times\left( nN-mN+m \right) \end{matrix}$ (S6)

This modification establishes a unique one-to-one mapping between the modulating frequency and lateral position, thereby eliminating conventional spatial ambiguities. The minimum frequency interval resulting from the 2D Bragg diffraction in this regime is denoted as $\delta f={f_{\mathrm{range}}}/{N\left( N-1 \right)}$.

Considering the base frequency $f_{0}$ carried by the probing illumination, the practical oscillation frequency takes the form of $f_{0}+{f_{\mathrm{range}}}/{N\left( N-1 \right)}\times\left( nN-mN+m \right)$, enabling common-path (coaxial) interference with the conserved zero-order beam $f_{0}$ for heterodyne holography. Ideally, complex-field reconstruction can be theoretically achieved using fast Fourier transformation (FFT), where each complex-field value reflected from the dynamic object is retrieved by the corresponding index of frequency tones. However, a significant challenge arises during data acquisition with coaxial heterodyne holography when using a single-pixel detector for direct intensity measurements, as this approach loses all phase information. Calculating complex-field retrieval with FFT from the temporal intensity signal measured by the single-pixel detector results in only side-band detection, with substantial crosstalk between bilaterally symmetric frequency tones.

Referring back to Eq. S6, a series of unique $f\left( x_{n},y_{m} \right)$ values still include numerous symmetric pairs based on the term $nN-mN+m$, leading to spectral aliasing, as illustrated in Figs. S3a and S3b. To resolve this, it is crucial to break the symmetry of the 2D frequency combs, marking the next advanced improvement of the FACE pattern. Returning to the original stipulation in Eq. S3, a rational modification involves adjusting the frequency locations $f_{\mathrm{int}}$ and $f_{\mathrm{end}}$, which were previously set to the same value for simplicity. By introducing a subtle shift $\delta f$ in the x direction (or the y direction), a discrepancy is created between $f_{x\_int}$ and $f_{y\_int}$ (also between $f_{x\_end}$ and $f_{y\_end}$). Beyond merely adjusting the frequency intervals, this discrepancy induces an offset for the updated second term $f_{x\_int}-f_{y\_int}$ as referenced in Eq. S5, introducing a subtle frequency shift $f_{\mathrm{shift}}$, which will be determined next. With this induced frequency offset, Eq. S6 can be further advanced:

$f\left( x_{n},y_{m} \right)=f_{0}+\delta f\times\left( nN-mN+m \right)\pm f_{\mathrm{shift}}$ (S7)

where the adjustable $\pm f_{\mathrm{shift}}$ introduces a subtle shift to prevent frequency overlap. A rational choice for $f_{\mathrm{shift}}$ will be further discussed in the practical determination of appropriate parameters in Supplement Note 6. The appropriate frequency shift $f_{\mathrm{shift}}$ to avoid any unintended overlaps should lie within a well-defined range, expressed as:

$\begin{matrix} f_{\mathrm{shift}}\in\left( f_{\mathrm{shift}}|-\delta f-f_{\mathrm{shift}}<0+f_{\mathrm{shift}}<\delta f-f_{\mathrm{shift}} \right) \\ \Longrightarrow f_{\mathrm{shift}}\in\left( f_{\mathrm{shift}}|-0.5\delta f<f_{\mathrm{shift}}<0.5\delta f \right) \end{matrix}$ (S8)

In this scenario, the updated minimum interval $\delta\hat{f}$ for FACE in Eq. S7 is derived as:

$\delta\hat{f}=\min\left( 2f_{\mathrm{shift}},\delta f-2f_{\mathrm{shift}} \right)$ (S9)

The hat notation represents the corresponding updated versions of the variables, and this applies to the rest of the definitions as well. To maximize $\delta\hat{f}$ for clearly distinguishing frequency combs in coherent detection, the optimal choice for $\hat{f}_{\mathrm{shift}}$ is $\hat{f}_{\mathrm{shift}}={\delta f}/4$, resulting in $\delta\hat{f}={\delta f}/2$. This advanced strategy effectively minimizes ambiguity and optimizes the retrieval process for FACE-SPCM. A detailed illustration of this concept is provided in Fig. S3c.

**Supplementary Note 5: Spatiotemporal calibration and phase contamination correction in heterodyne holography**

This section illustrates the complex-field reconstruction $O\left( x_{n},y_{m} \right)\propto A\left( x_{n},y_{m} \right)\exp\left( i\phi\left( x_{n},y_{m} \right) \right)$ enabled by the FFT of the temporal intensity signal $I\left( t \right)$. Based on the beat-frequency oscillation representation, $\hat{I}\left( t \right)$ is constructed with frequency tones $f\left( x_{n},y_{m} \right)$ as:

$\hat{I}\left( t \right)|_{f\left( x_{n},y_{m} \right)}=E_{R}A\left( x_{n},y_{m} \right)\cos\left( 2\pi f\left( x_{n},y_{m} \right)t+\phi\left( x_{n},y_{m} \right) \right)$ (S10)

Here, $\hat{I}\left( t \right)|_{f\left( x_{n},y_{m} \right)}$ represents the beat-frequency oscillation between $f\left( x_{n},y_{m} \right)$ and the base frequency $f_{0}$, with $E_{R}$ indicating the energy modality of the reference beam. Ideally, FFT can accurately separate the corresponding heterodyne holography signals, simultaneously extracting both amplitude $A\left( x_{n},y_{m} \right)$ and phase $\phi\left( x_{n},y_{m} \right)$. However, in practice, establishing the beat frequency with the zero-order beam $f_{0}$ cannot ensure a consistent start point $t_{0}$ for subsequent acquisition. The term $2\pi f\left( x_{n},y_{m} \right)t_{0}+\phi\left( x_{n},y_{m} \right)$ introduces variable phase representations due to frequency oscillation contamination, denoted by $2\pi f\left( x_{n},y_{m} \right)t$. This issue, identified as the first type of phase disruption, is visually labeled as “Frequency oscillation” in Fig. S4a.

This issue vanishes when $t_{0}=0$. Due to the consistent interval of discrepant frequency tones, the synthesized heterodyne holography exhibits periodicity, allowing phase reversion at a specific time $t_{0}=\hat{t}$. For all frequency tones $f\left( x_{n},y_{m} \right)$, $\hat{t}$ is derived from the least common multiple of all frequency intervals as $\hat{t}=1/{\hat{f}_{\mathrm{shift}}}$, satisfying:

$2\pi f\left( x_{n},y_{m} \right)\hat{t}\equiv2h\pi$ (S11)

Here, $h$ is an integer, and $\hat{t}$ represents the minimum period that satisfies $h$ multiples of the phase $2\pi$ in the synthesized heterodyne holography referenced in Eqs. S7–S9, ensuring accurate reconstruction of $\phi\left( x_{n},y_{m} \right)$. As shown in Fig. S4a, the phase error $2\pi f\left( x_{n},y_{m} \right)t$ induced by discrepant frequency tones reverts to the same state over time $\hat{t}$. For dynamic scenes, this parameter $\hat{t}$ is analogous to the acquisition time. Although temporal evolution is continuous, sampling this temporal evolution from the dynamic scene into slices with minimum resolvable $\hat{t}$ spacing yields $O\left( x_{n},y_{m},l\hat{t} \right)\approx O\left( x_{n},y_{m},l\hat{t}+\delta t \right)\propto A\left( x_{n},y_{m},l\hat{t} \right)\exp\left( i\phi\left( x_{n},y_{m},l\hat{t} \right) \right)$ at the *l*-th temporal slice. This is depicted in the first row of Fig. S4a, neglecting the subtle variation of an ultrashort moment $\delta t$ within $\hat{t}$. An improved representation of beat-frequency oscillation for phase retrieval is shown as follows:

$\begin{matrix} \hat{I}\left( t \right)|_{f\left( x_{n},y_{m} \right)}=E_{R}A\left( x_{n},y_{m},l\hat{t} \right)\cos\left( 2\pi f\left( x_{n},y_{m} \right)\left( t+l\hat{t} \right)+\phi\left( x_{n},y_{m},l\hat{t} \right) \right) \\ \Longrightarrow E_{R}A\left( x_{n},y_{m},l\hat{t} \right)\cos\left( 2\pi f\left( x_{n},y_{m} \right)t+\phi\left( x_{n},y_{m},l\hat{t} \right) \right) \end{matrix}$ (S12)

This represents the minimum acquisition time needed for simultaneous reconstruction, corresponding to the complete phase reversion of $2\pi$.


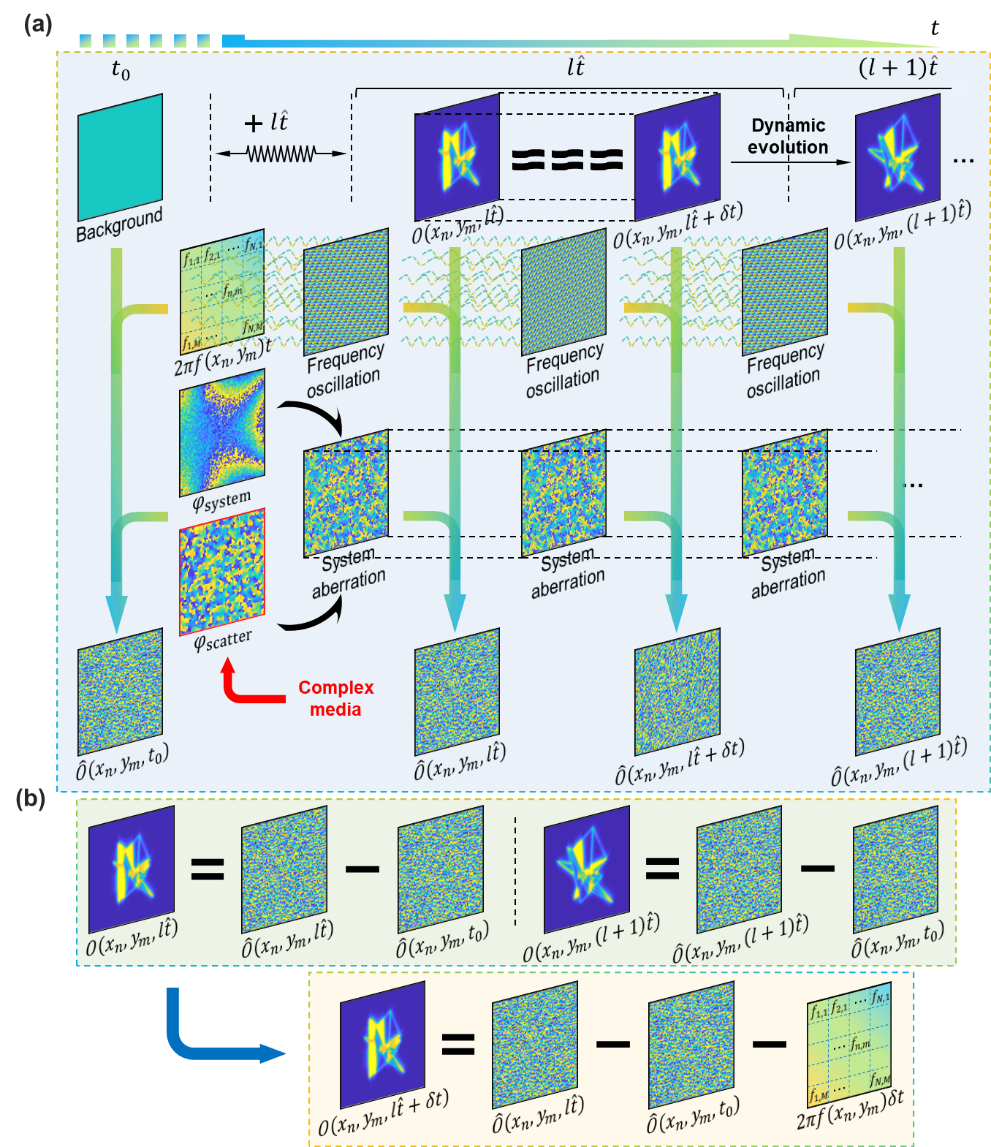


**Figure S4 | Comprehensive calibration process for complex-field imaging reconstruction.** (a) In a dynamic, time-varying complex-field scenario, phase disruptions caused by inherent system aberrations obscure the imaging information of the target scene. These phase aberrations include frequency oscillations from the FACE scheme, structural aberrations from the experimental setup, and optical scattering from complex media. (b) The calibration procedure using a blank background is employed to eliminate disruptions from the various aberrations mentioned. A potential application for achieving ultrahigh temporal resolution is also illustrated.

Figure S4a also highlights imperfections in the digital construction of the FACE pattern and the physical setup of the optical imaging system. Imperfections in the 2D Bragg diffractions, caused by OFDM radio-frequency signals with finite sampling rates and lack of synchronization, introduce phase distributions $\varphi_{\mathrm{FACE}}\left( f\left( x_{n},y_{m} \right) \right)\Longrightarrow\varphi_{\mathrm{FACE}}\left( x_{n},y_{m} \right)$ within the FACE pattern. Additionally, any unachievable details, as discussed in Supplementary Note 2, resulting from imperfect implementations can induce further structural phase aberrations $\varphi_{\mathrm{aber}}\left( x_{n},y_{m} \right)$ on the object. These imperfections combine to form system-induced aberrations:

$\varphi_{\mathrm{system}}\left( x_{n},y_{m} \right)=\varphi_{\mathrm{FACE}}\left( x_{n},y_{m} \right)+\varphi_{\mathrm{aber}}\left( x_{n},y_{m} \right)$ (S13)

The beat-frequency signal is subsequently revised as:

$\hat{I}\left( t \right)|_{f\left( x_{n},y_{m} \right)}=E_{R}A\left( x_{n},y_{m},l\hat{t} \right)\cos\left( 2\pi f\left( x_{n},y_{m} \right)t+\varphi_{\mathrm{system}}\left( x_{n},y_{m} \right)+\phi\left( x_{n},y_{m},l\hat{t} \right) \right)$ (S14)

Using FFT, the retrieved complex-field information is:

$E_{R}A\left( x_{n},y_{m},l\hat{t} \right)\exp\left( i(\varphi_{\mathrm{system}}\left( x_{n},y_{m} \right)+\phi\left( x_{n},y_{m},l\hat{t} \right)) \right)$ (S15)

where the term $\varphi_{\mathrm{system}}\left( x_{n},y_{m} \right)+\phi\left( x_{n},y_{m},l\hat{t} \right)$ highlights the obscurity introduced by unknown system-induced aberrations during the retrieval process.

In scenarios where the system images dynamic targets through complex media, light propagation through scattering events leads to intricate interactions, reflecting a linear system with Gaussian transmission characteristics between the target plane and the detector. The spectral-encoded projection through the objects undergoes unpredictable complex-field responses $R_{\mathrm{scatter}}=A_{\mathrm{scatter}}\left( x_{n},y_{m} \right)\exp\left( i\varphi_{\mathrm{scatter}}\left( x_{n},y_{m} \right) \right)$. This is analogous to heterodyne holography obtained by a photodetector. The revised form $\hat{O}\left( x_{n},y_{m},l\hat{t} \right)$ through FFT is:

$\begin{matrix} \hat{O}\left( x_{n},y_{m},l\hat{t} \right)=E_{R}A_{\mathrm{scatter}}\left( x_{n},y_{m} \right)O\left( x_{n},y_{m},l\hat{t} \right)\exp\left( i(\varphi_{\mathrm{system}}\left( x_{n},y_{m} \right)+\varphi_{\mathrm{scatter}}\left( x_{n},y_{m} \right) \right) \\ =E_{R}A_{\mathrm{scatter}}\left( x_{n},y_{m} \right)A\left( x_{n},y_{m},l\hat{t} \right)\exp\left( i(\varphi_{\mathrm{system}}\left( x_{n},y_{m} \right)+\varphi_{\mathrm{scatter}}\left( x_{n},y_{m} \right)+\phi\left( x_{n},y_{m},l\hat{t} \right)) \right) \end{matrix}$ (S16)

This phase aberration, jointly induced by the terms $\varphi_{\mathrm{system}}\left( x_{n},y_{m} \right)+\varphi_{\mathrm{scatter}}\left( x_{n},y_{m} \right)$ from the practical system, represents the second phase disruption, which is visually labeled as “System aberration” in Fig. S4a.

To resolve this ambiguity, an optional calibration process is essential. The calibration is performed without dynamic scenes, starting with $O\left( x_{n},y_{m},t_{0} \right)=1$ for a transparent geometry. This process captures all system-induced aberrations for a global reference, recording as:

$\hat{O}\left( x_{n},y_{m},t_{0} \right)=E_{R}A_{\mathrm{scatter}}\left( x_{n},y_{m} \right)exp(i(\varphi_{\mathrm{system}}\left( x_{n},y_{m} \right)+\varphi_{\mathrm{scatter}}\left( x_{n},y_{m} \right)))$ (S17)

By subtracting these quantified aberrations, the complex field of dynamic objects can be retrieved without ambiguity. The elimination of system-induced aberrations and scattering effects caused by embedded complex media through calibration is illustrated in the pale-green box in Fig. S4b.

In addition, this calibration is crucial for addressing unpredictable variations in real-time tracking, enabling ultrafast analysis of complex-field evolution in dynamic scenarios. The disrupting term $2\pi f\left( x_{n},y_{m} \right)t_{0}$ functions as a temporal domain aberration. Without strict synchronization to $t_{0}=0$, the calibration process aids in complex-field reconstruction of dynamic scenes at the sampling interval $\hat{t}$. For continuous offline processing, the acquisition time $\hat{t}$ is minimized to the limit defined by the sampling rate of the digital acquisition card (DAC), given by $\delta t=1/{S_{\mathrm{DAC}}}$. Assuming that the ultrafast evolution $\phi\left( x_{n},y_{m},l\hat{t}+\delta t \right)$ is indistinguishable by the system, the iterative beat-frequency oscillation at $f\left( x_{n},y_{m} \right)$ can be expressed as:

$\hat{I}\left( t \right)|_{f\left( x_{n},y_{m} \right)}=E_{R}A\left( x_{n},y_{m},l\hat{t}+\delta t \right)\cos\left( 2\pi f\left( x_{n},y_{m} \right)\left( t+l\hat{t}+\delta t \right)+\phi\left( x_{n},y_{m},l\hat{t}+\delta t \right) \right)$ (S18)

Much shorter than the periodicity, the volatile term $2\pi f\left( x_{n},y_{m} \right)\delta t$, although not removable by constant calibration, permits intuitive signal processing and ultrafast analysis of continuous temporal evolution. This concept is illustrated in the pale-orange box of Fig. S4b.

**Supplementary Note 6: Techniques for optimizing spatial coherence in coaxial heterodyne holography**

Focusing on coherent measurement, this section explores the internal mechanism for establishing coaxial heterodyne holography, with emphasis on the role of a specialized optical grating. Unlike traditional holography systems that use a Mach–Zehnder interferometer, the FACE-SPCM system relies on the interaction between the ejection angle and frequency-comb encoding during Bragg diffraction, which influences the spatial angular spectrum for free-space interference. When targeting the central component $f_{\mathrm{center}}$ of the FACE pattern and the main illumination with base frequency $f_{0}$ are targeted, a well-defined deflecting angle $\Delta\theta_{\mathrm{center}}$ is derived from Eq. S2. Assuming quasi-plane wave illumination, their interference produces stable fringes across a transverse section, ensuring strong coherence. Consequently, the fringe period $x_{T}$ is given by:

$x_{T}=\lambda/{\sin\Delta\theta_{\mathrm{center}}}$ (S19)

Here, the fringe period $x_{T}$ is inversely proportional to the deflection angle $\Delta\theta_{\mathrm{center}}$. A large deflection angle results in a significant reduction of $x_{T}$, creating an unexpectedly dense fringe pattern. Theoretically, this would pose no issues if detection were performed by an ideal single-pixel “point” detector. However, in practice, the photodetector chip has a finite area and cannot be reduced to a perfect “point,” leading to non-negligible deterioration in energy collection due to its finite size.

Mathematically, the fringe profile along a direction $r$ is given by $a+b\cos\left( 2\pi r/{x_{T}} \right)$, where the ratio $b/a$ closely relates to the interference contrast. Due to space-time duality, this contrast also affects the coherence of detected heterodyne holography $\hat{I}\left( t \right)|_{f\left( x_{n},y_{m} \right)}$. With a considerably small period $x_{T}$ and a finite sensing size $r_{\mathrm{PD}}$, the collected intensity by a scanning photodetector is:

$\begin{matrix} I\left( r \right)=\int_{r-{r_{\mathrm{PD}}}/2}^{{r+r_{\mathrm{PD}}}/2} a+b\cos\left( 2\pi{r^{'}}/{x_{T}} \right)dr^{'} \\ \Longrightarrow ar_{\mathrm{PD}}+b\frac{x_{T}}{\pi}\sin\left( \pi{r_{\mathrm{PD}}}/{x_{T}} \right)\cos\left( 2\pi r/{x_{T}} \right) \end{matrix}$ (S20)

This expression updates the interference contrast from $b/a$ into $\left( b/a \right)\mathrm{sinc}\left( {r_{\mathrm{PD}}}/{x_{T}} \right)$. With a smaller $x_{T}>0$ and a finite $r_{\mathrm{PD}}>0$, an increasing ${r_{\mathrm{PD}}}/{x_{T}}$ would result in a decreasing interference contrast, thereby deteriorating the subsequent heterodyne holography.

To enhance interference contrast and improve the signal-to-noise ratio (SNR), minimizing deflection angles, especially those interacting with the middle branch of the FACE pattern, is crucial. Introducing an optical grating to converge deflected illuminations at smaller angles is an effective strategy. For a sinusoidal periodic grating with grating constant $d$, the first diffraction angle $\Delta\theta_{\mathrm{grating}}$ at $k=1$ is given by: $d\sin\left( \Delta\theta_{\mathrm{grating}} \right)=k\lambda$. Aligning the reference beam with the central branch direction such that $\Delta\theta_{\mathrm{grating}}\approx\Delta\theta_{\mathrm{center}}$, the grating design follows:

$d=\lambda/{\sin\left( {\lambda\Delta f_{\mathrm{center}}}/V \right)}$ (S21)

As shown in Fig. S5, the reference beam splits into three components ($k=0,\pm1$). For the FACE illumination, only the central branch ($k=0$) is considered, as other similar deflections are neglected for simplification. After passing through a well-designed grating, the two relevant illuminations ($k=+1$ from the reference beam and $k=0$ from the FACE pattern) converge, ensuring strong coherence.


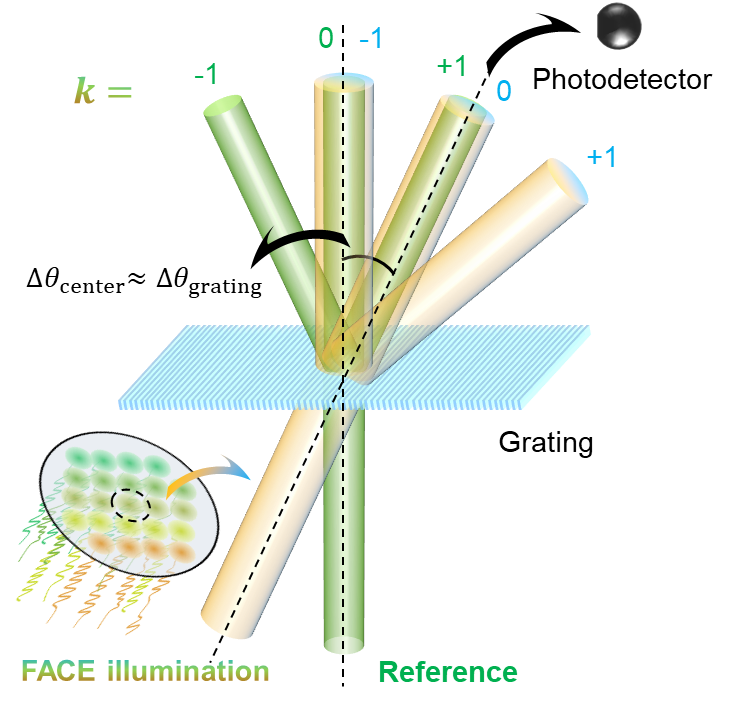


**Figure S5 | Schematic diagram of optical grating for beam convergence.** The FACE pattern and the unmodulated reference beam undergo diffraction, splitting into three components. The heterodyne holography collected by the photodetector is formed by the 0th-order component of the FACE pattern and the 1st-order component of the reference beam.

A 4*f* imaging system can adjust not only the field of view (FoV) but also the deflection angle. The scalar relationship imposed on the deflection angles, beyond spatial scaling, suggests an inverse relationship between the deflection angle and spatial scaling factor. Therefore, a well-designed 4*f* imaging system can mitigate this type of deterioration, though at the expense of reduced energy density. For a prior imaging scheme, detailed as Imaging Scheme 1 in the following Supplementary Note 7, a 50 lp mm^-1^ optical grating and a rear lens with a 25 mm focal length in the 4*f* system are used for final acquisition.

**Supplementary Note 7: Configurations for specialized dynamic scene imaging**

This section outlines the imaging parameters required to establish an optimal configuration for ultrahigh-throughput FACE-SPCM. Given the critical role of imaging speed, we start with the stipulated $\hat{t}$ for the FACE scheme, which determines the imaging FPS as $F_{\mathrm{imaging}}=1/\hat{t}$. As discussed in Supplementary Note 5, $\hat{t}=1/{\hat{f}_{\mathrm{shift}}}$ implies that $F_{\mathrm{imaging}}=\hat{f}_{\mathrm{shift}}$ when $\hat{f}_{\mathrm{shift}}$ is optimally designed to maximize the minimum interval $\delta\hat{f}$. By setting $\hat{f}_{\mathrm{shift}}={\delta f}/4$ and $\delta\hat{f}={\delta f}/2$, we can reverse-engineer $\delta f$ from Eq. S7.

The interval $\delta f={f_{\mathrm{range}}}/{N\left( N-1 \right)}$ relates directly to the bandwidth of the AOD and photodetector. For our practical system, the AODs modulate the frequency shifting within 61–91 MHz, limiting $f_{\mathrm{range}}$ to approximately 30 MHz. The single-pixel detector (APD130C, Thorlabs) detects beat-frequency signals around 60 MHz but has a finite bandwidth of 50 MHz, bringing a severe situation of spectrum overlapping reversely for the side-band detection within an entire signal spectrum $\left( -f_{\mathrm{range}},f_{\mathrm{range}} \right)$. To avoid this, it necessitates a modulation bandwidth within 25 MHz ($f_{\mathrm{range}}<25\mathrm{MHz}$).

Assuming central modulating frequencies of 76 MHz for both AODs, the FACE-SPCM system with an FPS of $F_{\mathrm{imaging}}=1,000$ Hz determines the frequency combs for both orthogonal AODs. The choice of 1,000 FPS would be an appropriate value. If too high, it will result in broader intervals and limit the finite frame size, while a slow FPS with a large frame size may deteriorate the imaging quality due to the limitations of the light source’s line-width noise. Accordingly, The derived parameters are $\delta f=4000$, $N=79$ with $\Delta f_{x}={f_{\mathrm{range}}}/\left( N-1 \right)$, $M=80$ with $\Delta f_{y}={f_{\mathrm{range}}}/\left( M-1 \right)$, and $f_{\mathrm{range}}\approx24\mathrm{MHz}$. The detailed configuration is:

$\left\{ \begin{matrix} \left\{ f_{x} \right\}=\left( -63.752\mathrm{MHz}:-316\mathrm{kHz}:-88.400\mathrm{MHz} \right)-1000 \mathrm{Hz} \\ \left\{ f_{y} \right\}=\left( +63.752\mathrm{MHz}:+312\mathrm{kHz}:+88.400\mathrm{MHz} \right) \end{matrix} \right.$ (S22)

Designing these frequency combs using the OFDM strategy requires integer multiples for efficient digital conservation. The greatest common factor is 316 kHz for $\left\{ f_{x} \right\}$ in the x-direction frequency combs and 312 kHz for $\left\{ f_{y} \right\}$ in the y-direction frequency combs, with a subtle shift $\hat{f}_{\mathrm{shift}}=1,000\mathrm{kHz}$. Considering an imaging lens with a 7.5-mm focal length, the comprehensive parameters for ultrafast imaging are detailed in Supplementary Table 3:

**Supplementary Tab. 3 Parameters configured by Imaging Scheme 1 for ultrafast SPCM**

| **Parameters** | **Reference values** |
| --- | --- |
| Imaging Scheme 1: ultrafast FACE-SPCM | |
| Material acoustic mode velocity ($V$) | TeO2 [S] – 650 m s^-1^ |
| Acousto-optic active area ($D_{\mathrm{beam}}$) | 5 × 5 mm |
| Frequency comb in AODx ($\left\{ f_{x} \right\}$) | -63.752 MHz : -316 kHz : -88.400 MHz - 1000 Hz |
| Frequency comb in AODy ($\left\{ f_{y} \right\}$) | +63.752 MHz : +312 kHz : +88.400 MHz |
| X-direction deflecting angles ($\left\{ \theta_{x} \right\}$) | -101.02 mrad : -500.74 μrad : -140.08 mrad |
| Y-direction deflecting angles ($\left\{ \theta_{y} \right\}$) | +101.02 mrad : +494.40 μrad : +140.08 mrad |
| Focus length of imaging lens ($f_{i}$) | 7.5 mm |
| Acousto-optic x-direction resolution ($R_{x}=f_{i}\delta\theta_{x}$) | 3.76 μm |
| Acousto-optic y-direction resolution ($R_{y}=f_{i}\delta\theta_{y}$) | 3.71 μm |
| Diffraction limitation ($R_{D}=0.61\lambda/{N.A.}$) | 1.99 μm |
| FoV ($f_{i}\Delta\theta_{x}\times f_{i}\Delta\theta_{y}$) | 293.28 × 293.28 μm |
| Final lateral resolution | 3.76 μm |

Numerical aperture (N.A.) = $\left( {D_{\mathrm{beam}}}/2 \right)/{\sqrt{\left( {D_{\mathrm{beam}}}/2 \right)^{2}+f_{i}^{2}}}$

Final lateral resolution = max ($R_{x}$, $R_{y}$, $R_{D}$)

In this setup, the spectral-encoded projection through the imaging lens generates a focusing array with focus sizes of approximately 1.99 μm, determined by the diffraction limit associated with the numerical aperture (N.A.) of a 7.5-mm-focal-length lens, making it smaller than the acousto-optic resolution ($R_{x}$ and $R_{y}$). Under these conditions, the final lateral resolution is determined by the largest of the three values.

For dynamic scenarios across various research fields, the flexibility in selecting the imaging lens and designing the FACE pattern provides a valuable opportunity to adapt the imaging structure. Conversely, for scenarios that require imaging of large-range changes or slow-evolving processes to capture more detailed information in the complex field domain, a rational trade-off between FPS, FoV, resolution, and beam quality should be considered. For broader applications, two alternative schemes for FACE-SPCM are detailed in Supplementary Table 4, which also support the proper observation windows for biological and chemical demonstration in the manuscript.

**Supplementary Tab. 4 Parameters configured by alternative Imaging Schemes 2 and 3 specialized for different scenarios**

| **Parameters** | **Reference values** |
| --- | --- |
| **Imaging Scheme 2:** specialized for a large FoV | |
| Frequency comb in AODx ($\left\{ f_{x} \right\}$) | -72.639 MHz : -256 kHz : -88.767 MHz - 1000 Hz |
| Frequency comb in AODy ($\left\{ f_{y} \right\}$) | +72.639 MHz : +252 kHz : +88.767 MHz |
| X-direction deflecting angles ($\left\{ \theta_{x} \right\}$) | -115.10 mrad : -405.66 μrad : -140.66 mrad |
| Y-direction deflecting angles ($\left\{ \theta_{y} \right\}$) | +115.10 mrad : +399.32 μrad : +140.66 mrad |
| Focus length of imaging lens ($f_{i}$) | 15.3 mm |
| Acousto-optic x-direction resolution ($R_{x}=f_{i}\delta\theta_{x}$) | 6.21 μm |
| Acousto-optic y-direction resolution ($R_{y}=f_{i}\delta\theta_{y}$) | 6.11 μm |
| Diffraction limitation ($R_{D}=0.61\lambda/{N.A.}$) | 3.90 μm |
| FoV ($f_{i}\Delta\theta_{x}\times f_{i}\Delta\theta_{y}$) | 391.23 × 391.23 μm |
| Final lateral resolution | 6.21 μm |
| **Imaging Scheme 3:** specialized for reduced imaging aberration | |
| Frequency comb in AODx ($\left\{ f_{x} \right\}$) | -71.5104 MHz : -126.4 kHz : -81.3696 MHz - 400 Hz |
| Frequency comb in AODy ($\left\{ f_{y} \right\}$) | +71.5104 MHz : +124.8 kHz : +81.3696 MHz |
| X-direction deflecting angles ($\left\{ \theta_{x} \right\}$) | -113.32 mrad : -200.30 μrad : -128.94 mrad |
| Y-direction deflecting angles ($\left\{ \theta_{y} \right\}$) | +113.32 mrad : +197.76 μrad : +128.94 mrad |
| Focus length of imaging lens ($f_{i}$) | 15.3 mm |
| Acousto-optic x-direction resolution ($R_{x}=f_{i}\delta\theta_{x}$) | 3.06 μm |
| Acousto-optic y-direction resolution ($R_{y}=f_{i}\delta\theta_{y}$) | 3.03 μm |
| Diffraction limitation ($R_{D}=0.61\lambda/{N.A.}$) | 3.90 μm |
| FoV ($f_{i}\Delta\theta_{x}\times f_{i}\Delta\theta_{y}$) | 239.37 × 239.37 μm |
| Final lateral resolution | 3.90 μm |

Numerical aperture (N.A.) = $\left( {D_{\mathrm{beam}}}/2 \right)/{\sqrt{\left( {D_{\mathrm{beam}}}/2 \right)^{2}+f_{i}^{2}}}$

Final lateral resolution = max ($R_{x}$, $R_{y}$, $R_{D}$)

It should be noted that the final lateral resolution differs from the acousto-optic resolution due to the combined effects of imaging diffraction limitation ($R_{D}$) and acousto-optic displacements ($R_{x}$ and $R_{y}$), as previously discussed. Experimental results for these alternative schemes are detailed in Supplementary Note 8.

**Supplementary Note 8: Additional experimental results for alternative imaging schemes**

Imaging Scheme 1, described in Supplementary Note 7, is used to produce the results detailed in the main text, including tests with a resolution test target and real-time dynamic imaging of oil-encapsulated water droplets. This section also presents additional imaging results obtained with the proposed FACE-SPCM using alternative schemes.


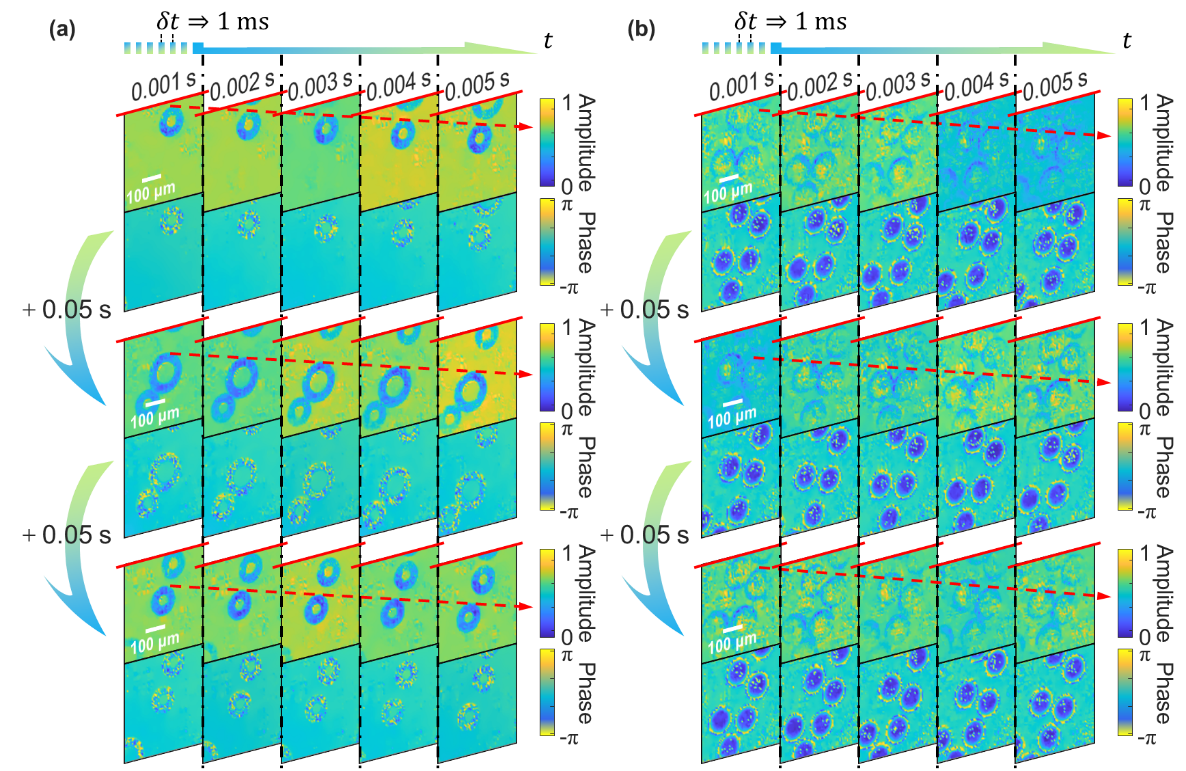


**Figure S6 | Experimental demonstration of microfluidics microscopy using Imaging Scheme 2.** (a) Temporal evolution of stable bubble production captured by the FACE-SPCM system in real time. (b) Temporal evolution of stable encapsulated droplets captured by the FACE-SPCM system in real time. The real-time streaming is sampled with ultrashort intervals of 1 ms and short intervals of 50 ms, demonstrating the system’s ability to track dynamic changes with high temporal resolution. Scale bar: 100 μm. Average CNR: 15.99.

Using the same microfluidic chip as described in the manuscript, Imaging Scheme 2 was tested. Figures S6a and S6b show time-varying sequences of liquid bubbles and stable oil-encapsulated water droplets with a reconstructed average CNR of 15.99. Designed for a larger FoV, Imaging Scheme 2 provides a zoomed-out perspective of droplets flowing through the microstructure.


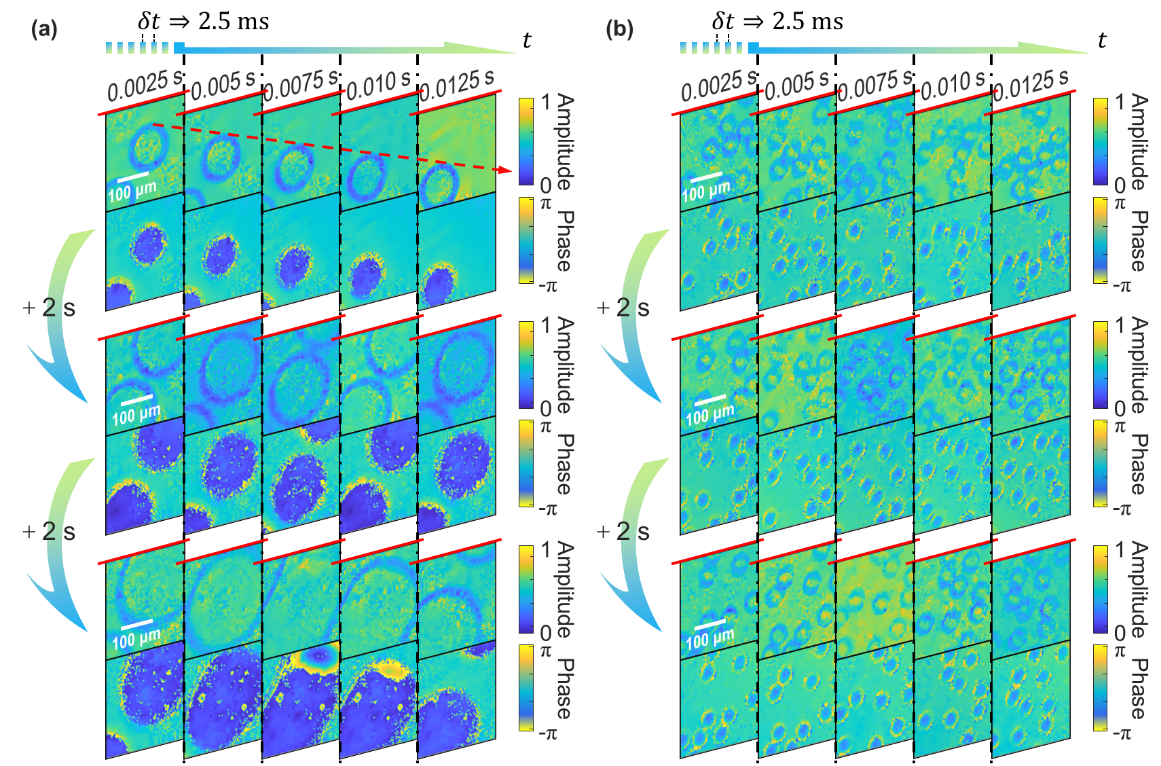


**Figure S7 | Experimental demonstration of microfluidics microscopy using Imaging Scheme 3.** (a) Temporal evolution of stable bubble production captured by the FACE-SPCM system in real time. (b) Temporal evolution of stable encapsulated droplets captured by the FACE-SPCM system in real time. The real-time streaming is sampled with ultrashort intervals of 2.5 ms and longer intervals of 2 s, showcasing the system’s ability to monitor dynamic processes with varied temporal resolutions. Scale bar: 100 μm. Average CNR: 19.02.

Imaging Scheme 3 was tested with a different microfluidic chip featuring a 200 μm droplet nozzle size. Figures S7a and S7b present time-varying sequences of complex fields with enhanced detail with a reconstructed average CNR of 19.02. Phase imaging reveals high contrast when bubbles form, attributed to the excessive channel width (over 300 μm), which causes significant refractive index differences between air and oil, rather than solely uneven distribution of droplet oil as previously thought. This observation is further supported by the results in Fig. S7b, where stable oil-encapsulated water droplets exhibit lower phase contrast due to the similar refractive indices of water and oil.

Notably, while Imaging Schemes 1 and 2 achieve superior temporal resolution (1,000 FPS), Imaging Scheme 3 offers higher CNR values due to its sparser frequency-comb configuration and reduced aberrations. However, we ultimately prioritized Imaging Scheme 1 for the main-text demonstrations, as it provides an optimal balance between CNR performance—sufficient for resolving critical biochemical details—and the functional requirements of real-time diagnosis, including high FPS and resolution. This systematic evaluation confirms that Imaging Scheme 1 effectively meets the dual demands of visualization clarity and temporal responsiveness, both of which are essential for dynamic biochemical monitoring.

**Supplementary Note 9: FACE-SPCM for microfluidics microscopy through scattering media**

To prove strong robustness, the FACE-SPCM system enables microfluidics microscopy through scattering media while consistently maintaining its FoV and resolution. The introduction of scattering media typically induces unpredictable responses due to scattering events, which can disrupt the accurate reconstruction of spectral-encoded information, as illustrated on the right side of Fig. S8a. As a proof of concept, we used a scattering medium composed of a 300-micron-thick polydimethylsiloxane substrate doped with 5% barium titanate particles. Remarkably, with an appropriate calibration procedure, the complications induced by scattering—such as information scrambling—could be effectively managed, allowing the system to perform high-fidelity imaging through scattering media. The calibration process tailored for scattering conditions is detailed in Supplementary Note 5. As shown in Fig. S8b, the phase images clearly capture the stable dynamic process of droplet generation across a series of scenes, even in the presence of scattering media. This result demonstrates the system’s robustness in overcoming light scattering challenges, broadening the potential applications of SPCM in biomedical and environmental sciences, where such conditions are prevalent.


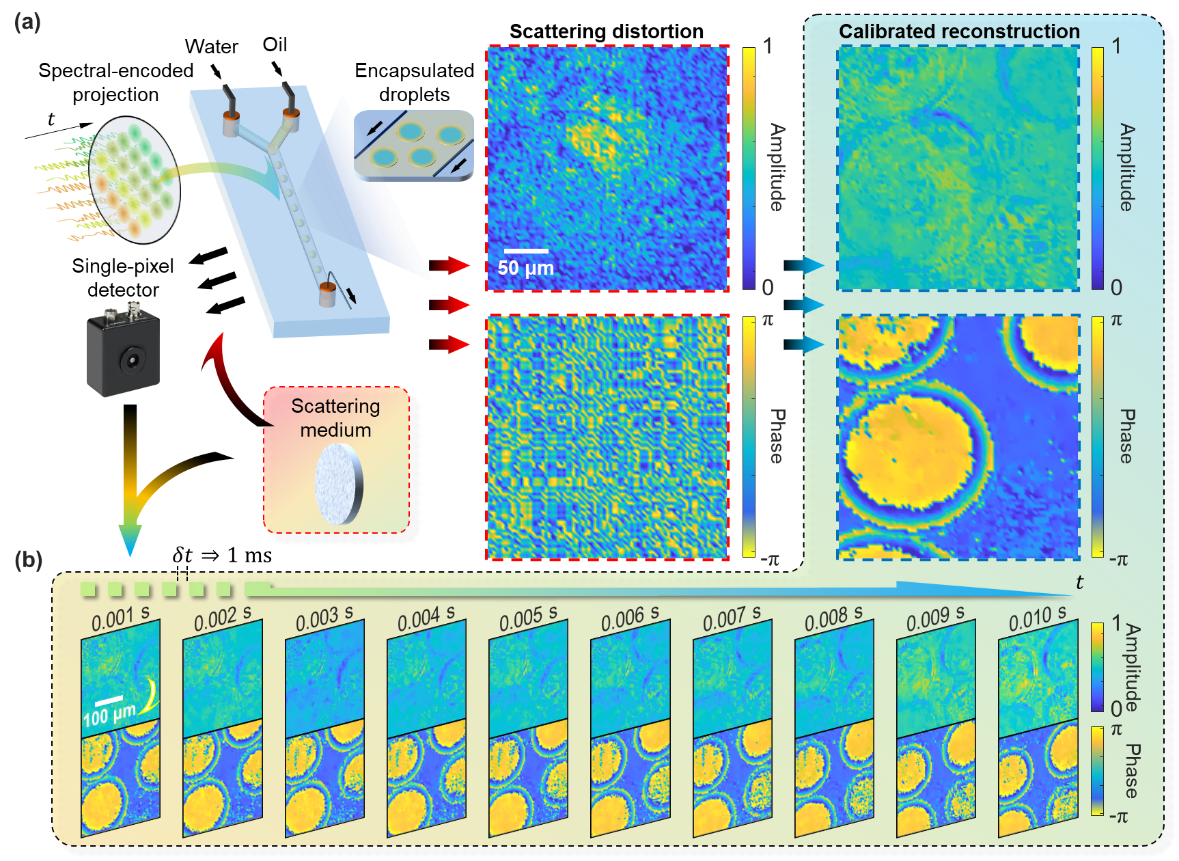


**Figure S8 | Experimental demonstration of microfluidics microscopy through scattering media.** (a) Initially, the FACE-SPCM system fails to reconstruct complex-field microscopy due to scattering aberrations introduced by the scattering medium, as shown in the middle image for visual demonstration. Scale bar: 50 μm. (b) After calibration, the system successfully reconstructs the complex-field microscopy of flowing encapsulated droplets, with holographic details clearly extracted. A series of complex-field images captured in temporal sequence further demonstrate the system’s capability for real-time monitoring through scattering media. Scale bar: 100 μm. Average CNR: 3.68.

Furthermore, to push the verification to a biological circumstance, we also evaluated the system’s imaging capability using biological tissue as the scattering medium, as illustrated in Fig. S9a. Without compensating for scattering effects, the holographic reconstruction, shown in Fig. S9b, exhibits significant image distortions. However, after applying the calibration process to compensate for scattering, the reconstructed images, presented in Fig. S9c, show substantial improvement. It is worth noting that due to the relatively strong absorption and instability of the tissue, the amplitude images exhibit a relatively low average CNR. Nevertheless, the phase images maintain good contrast, enabling clear identification of dynamic droplets.

**
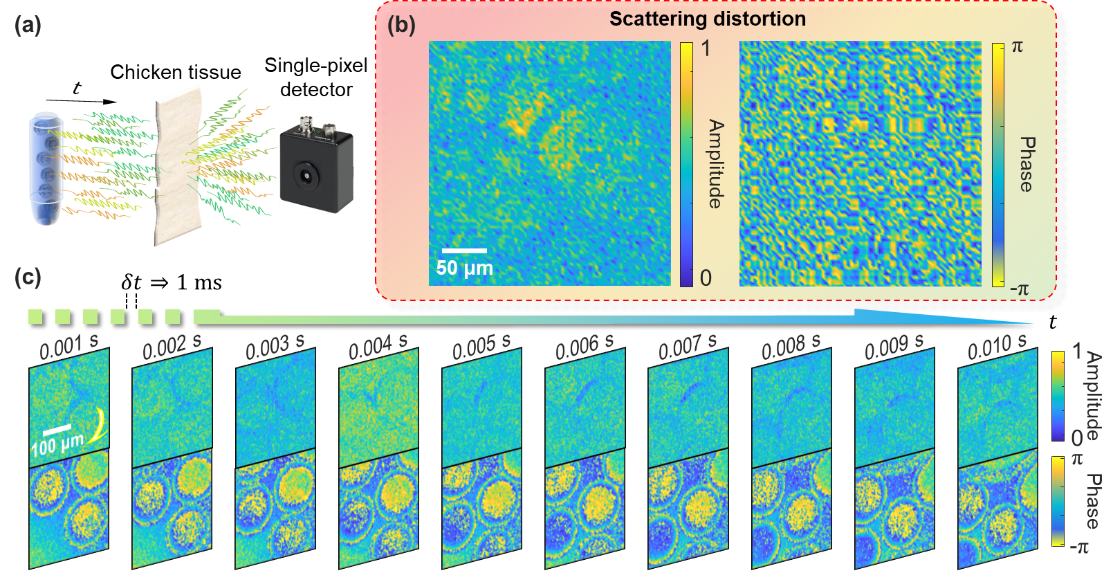
**

**Figure S9 | Experimental demonstration of microfluidics microscopy through chicken breast tissue.** (a) Schematic illustration of the microfluidic imaging setup with a 500-μm-thick chicken tissue slice as the scattering medium. (b) Without calibration, the FACE-SPCM system fails to reconstruct complex-field microscopy due to tissue-induced scattering. Scale bar: 50 μm. (c) After calibration, a series of complex-field images captured in temporal sequence further demonstrate the system’s capability for real-time monitoring through chicken breast tissue. Scale bar: 100 μm. Average CNR: 2.37.

**Supplementary Note 10: Effect of laser linewidth on imaging retrieval of FACE-SPCM**

Accurate complex-field reconstruction relies on the clear identification of frequency-comb information through FFT. Any noise that overlaps with neighboring frequency tones can cause severe crosstalk, reducing SNR and complicating retrieval. Since the expanding 2D frequency-comb array is derived from the same laser, the commonly stretched linewidth $\Delta f$ induced by phase drift noise is a critical factor contributing to spectral aliasing. This highlights our emphasis on maintaining coherence in this work. Given the fine structure of the frequency comb, a more stringent constraint on the laser’s linewidth is required, making its minimization crucial to ensure optimal performance.

This section aims to quantitatively assess how laser linewidth affects the accuracy of complex-field reconstruction in FACE-SPCM. Drawing inspiration from a phase-diffusion model tailored for laser spectral linewidth [12, 13], we approximate the impact using a Lorentzian profile to quantify interference during the practical retrieval process. A theoretical framework is then developed to evaluate the linewidth $\Delta f$ of the continuous wave (CW) laser, focusing on the temporal interference dynamics of heterodyne holography, rather than depending solely on empirical generalizations in the frequency domain.

According to the phase-diffusion model, the temporal illumination field can be expressed as:

$E\left( t \right)=E_{0}\exp\left( i2\pi f_{0}t+i\phi_{\mathrm{noise}}\left( t \right) \right)$ (S23)

where $E_{0}$ is the amplitude, $f_{0}$ is the base frequency, and $\phi_{\mathrm{noise}}\left( t \right)$ is a zero-mean time-varying fluctuation. Unlike typical Gaussian distributions, $\phi_{\mathrm{noise}}\left( t \right)$ in the phase-diffusion model evolves through accumulation from another defined random fluctuation $V_{r}\left( t \right)$. This relationship can be mathematically represented as:

$\phi_{\mathrm{noise}}\left( t \right)=2\pi\int_{-\infty}^{t} V_{r}\left( t^{'} \right)dt^{'}$ (S24)

The instantaneous frequency $f_{i}\left( t \right)$ at the moment $t$ is given by:

$f_{i}\left( t \right)=f_{0}+V_{r}\left( t \right)=f_{0}+\frac{1}{2\pi}\frac{d\phi_{\mathrm{noise}}}{dt}$ (S25)

Here, $V_{r}\left( t \right)$ is Gaussian white noise with zero mean $\mathcal{N}\left( 0,\sigma^{2} \right)$. This implies that, at any given moment, the instantaneous frequency $f_{i}\left( t \right)$ resulting from $V_{r}\left( t \right)$ can be treated as a Gaussian frequency drifting around $f_{0}$. The variance $\sigma^{2}$ of $V_{r}\left( t \right)$, modeled by laser linewidth $\Delta f$ and acquisition bandwidth $S_{\mathrm{DAC}}$, is given by:

$\sigma^{2}={\Delta fS_{\mathrm{DAC}}}/{2\pi}$ (S26)

Figure S10a illustrates the situation of different spectral linewidths determined by the phase-diffusion model, validating the relationship between phase noise and linewidth. For our CW laser (DHNL 1030.10-50-P-N-M-FA) with a linewidth of $\Delta f=3 \mathrm{kHz}$ and a sampling bandwidth of $S_{\mathrm{DAC}}=50 \mathrm{MHz}$, the 2D FACE pattern contains over 6000 tones with $\delta f=2000 \mathrm{Hz}$ after side-band detection.


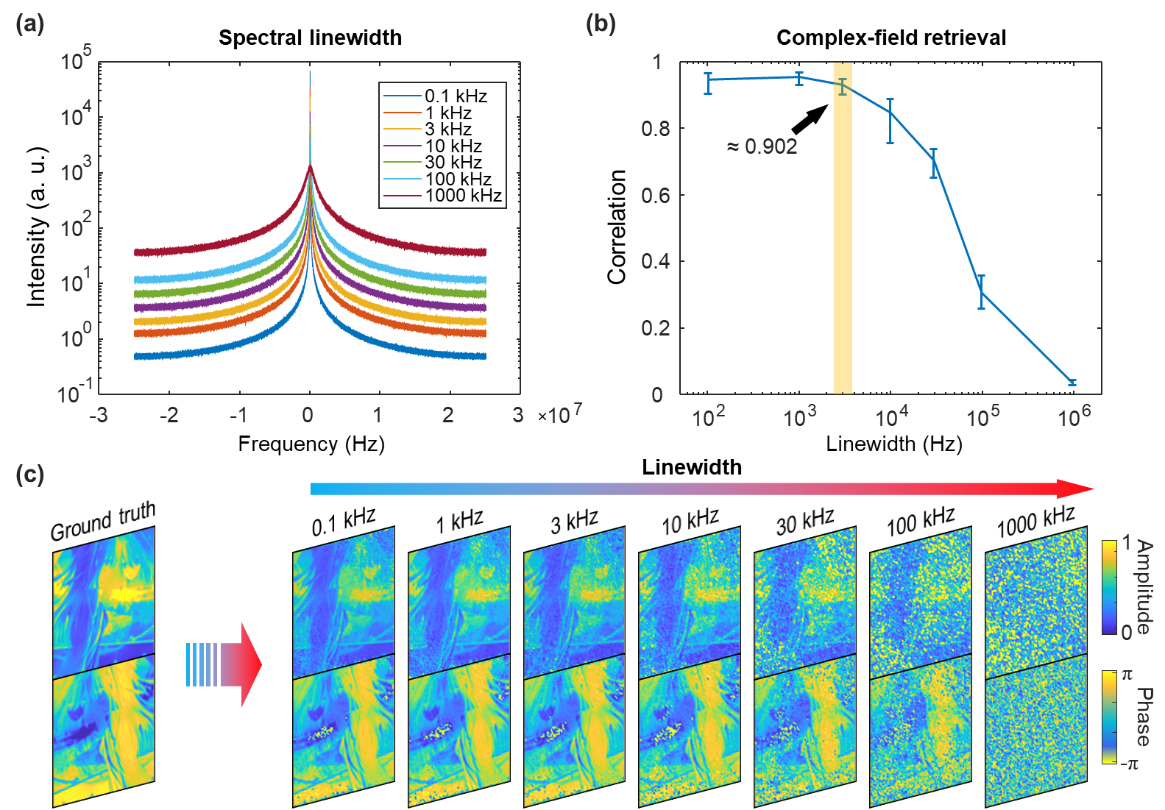


**Figure S10 | Simulation of laser linewidth effects on FACE-SPCM.** (a) Simulation of spectrum broadening induced by different laser linewidths due to phase drifting noise. (b) Correlation of complex-field retrieval plots as a function of varying laser linewidths in the simulation, demonstrating optimal performance at 3 kHz for the practical case. Error bar: standard deviations of 50 independent realizations. (c) Visual comparison of complex-field retrieval under different laser linewidths against the ground truth. The laser linewidths used in the simulations are [0.1 kHz, 1 kHz, 3 kHz, 10 kHz, 30 kHz, 100 kHz, 1000 kHz].

To validate the feasibility of the FACE-SPCM system, numerical simulations of FACE-SPCM were conducted with varying laser linewidths. Figure S10b shows the correlation of complex-field reconstruction as a function of laser linewidth, repeated 50 times. The practical laser linewidth of 3 kHz used for the FACE-SPCM system is highlighted in a pale-yellow box, demonstrating a high correlation (over 0.9), which strongly supports the practical applicability of the FACE-SPCM system. This performance is primarily attributed to the innovative coaxial coherent measurement design, which reduces optical path differences and mitigates phase noise disturbances from the laser linewidth. For visualization, Fig. S10c compares the retrieval performance for different linewidths, with the ground truth shown on the left side.

**Supplementary Note 11: Preparation of microfluidic devices for dynamic scenes**

In the experimental investigation of dynamic complex-field scenarios, all microfluidic devices are fabricated from polydimethylsiloxane (PDMS) and follow a consistent design with dual inlets and a single outlet. However, specific devices are uniquely tailored for generating encapsulated droplets or for mixing chemical solutions. As shown in Fig. S11, the microstructures of the microfluidic devices used for generating encapsulated droplets and mixing chemical solutions are depicted with distinct details, highlighting the flow direction and observation regions.


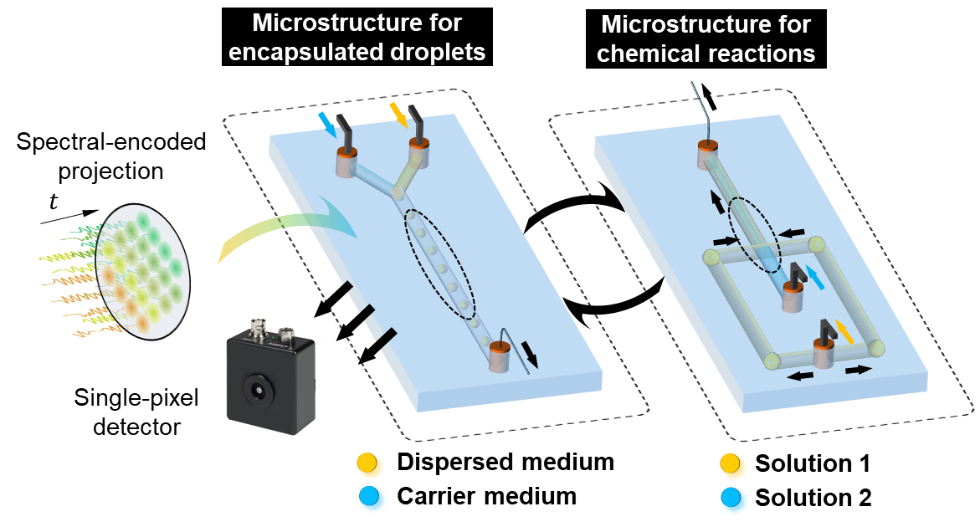


**Figure S11 | Microstructure used in FACE-SPCM’s microfluidics microscopy and chemical monitoring.** The first microstructure is designed for generating encapsulated droplets, intended for potential biological applications. The second microstructure is designed for mixing chemical solutions, aimed at potential chemical applications.

For the first setup, the “Results” section and Supplementary Notes 8 and 9 describe several microfluidic implementations using a microfluidic device with the first microstructure (PDMS-FF, FluidicLab), equipped with a droplet nozzle for stable droplet production. The device has two inlets for injecting the dispersed medium and carrier medium. The experiments shown in Fig. 3, as well as in Figs. S6, S8, and S9, utilize a microfluidic chip with a 100 μm droplet nozzle, while the data in Fig. S7 are obtained using a 200 μm nozzle. A larger droplet nozzle results in a proportionally wider channel; for instance, a 100 μm nozzle corresponds to a channel width of approximately 300 μm, while a 200 μm nozzle corresponds to a channel width of 500 μm.

For the second structure, a different microfluidic device (K0008, DXfluidics) is used to evaluate the capability of monitoring chemical reactions within transparent geometries. This device enables the mixing of different solutions to detect chemical reactions. It has two inlets, one for each type of solution, allowing observation of chemical reactions occurring at the confluence corner. To ensure a fair comparison, experiments involving acid-base neutralization (Fig. 4) and alcohol dilution (Fig. 5) are conducted using the same microfluidic chip, which has a channel width of approximately 250 μm and is specifically designed without a droplet nozzle.

**Supplementary Note 12: FACE-SPCM for imaging living microorganisms**

We expanded the application of the FACE-SPCM system to live biological observations, specifically focusing on imaging live paramecia in dynamic motion. Paramecium samples were placed on microscope slides with minimal preparation, as detailed in the “Methods” section, to ensure the organisms remained active and viable for dynamic observation. The system enabled us to capture intricate details of paramecia movement and behavior, providing valuable insights into their physiological processes and interactions. The ability to document these microorganisms’ activities across various time scales—from milliseconds to seconds—demonstrates the system’s flexibility and robustness in meeting diverse observational needs. Figure S12 shows a time-lapse sequence of amplitude and phase images, each annotated with the corresponding elapsed time, revealing both the rapid movements of paramecia and their longer-term interactions and behaviors. The temporal sampling can also be optimized for an appropriate observation window of paramecia movement, as described in Supplementary Note 7. The system’s capability to capture both amplitude and phase information enhances the depth of analysis, offering a more comprehensive understanding of biological dynamics. Supplementary Movie S4, slowed down by a factor of eight, further illustrates the dynamic observation capabilities of the FACE-SPCM system, highlighting its potential to advance real-time biomedical imaging.


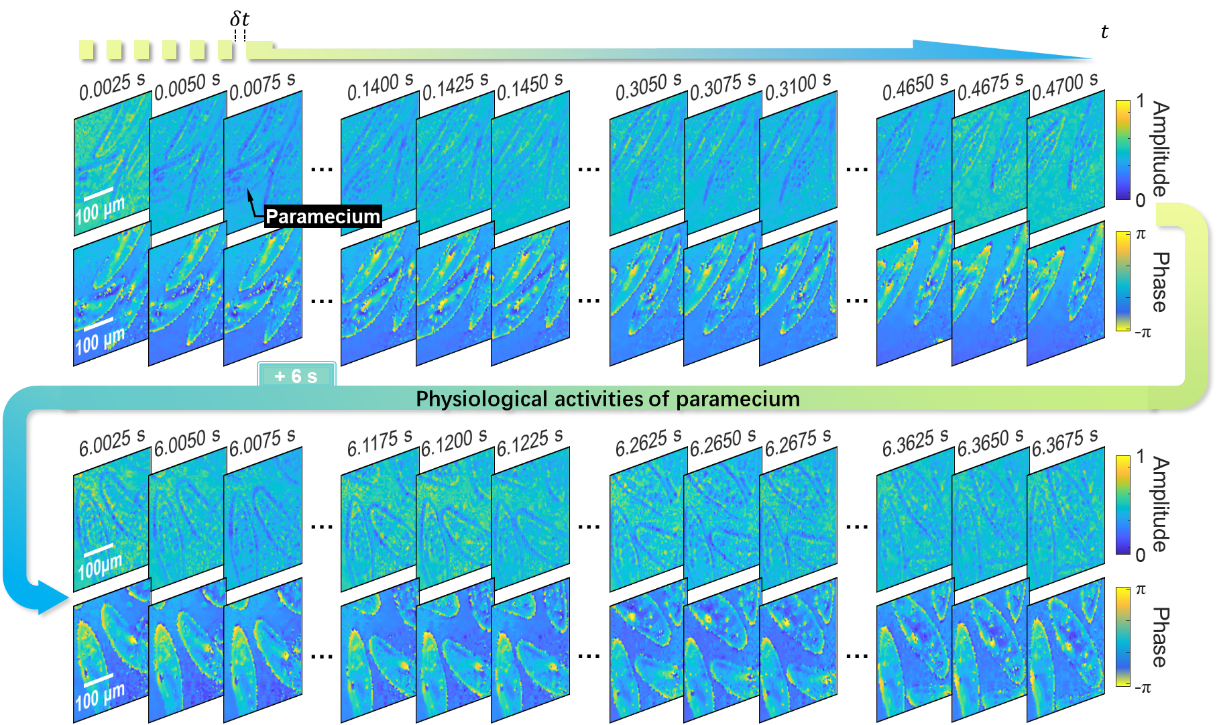


**Figure S12 | Experimental demonstration of FACE-SPCM in living microorganisms.** A group of living paramecia was selected for observation, and placed on a microscope slide with minimal preparation. The physiological activities of the paramecia were investigated in real-time, capturing both amplitude and phase details across various temporal scales. This demonstration confirms the system’s capability for real-time monitoring over ultrashort, short, and long intervals. Scale bar: 100 μm. Average CNR: 6.90.

**Supplementary Note 13: Quantitative analysis of the trade-offs between frame size and FPS**

This section explains why frame size and FPS are inherently constrained in FACE-SPCM. Specifically, a frame size of $N\times M$ requires identifying the same number of frequency combs in the spectrum. Given a fixed acousto-optic modulation bandwidth $f_{\mathrm{range}}$, the frequency separation between adjacent combs would prioritize a uniform sampling guess with reasonable supposition of ${f_{\mathrm{range}}}/{N\times M}$. According to Supplementary Note 4, the discussion of spatial ambiguities in FACE scheme necessitates a nontrivial design for orthogonal frequency tones, indicating that the exact frequency separation between adjacent components is not always uniform. At this meanwhile, a reasonable choice of the frame size stipulates the values of $N$ and $M=N+1$ for simplicity, assuming that the minimum resolvable frequency separation appears slight misalignments with ${f_{\mathrm{range}}\left( M-N \right)}/{N\times M}={f_{\mathrm{range}}}/{N\times M}$, which closely align with the expected supposition.

To acquire the frequency spectrum, the 1D signal must be measured over a duration *T*. According to the principles of Fourier transformation, *T* should be at least the inverse of the minimal frequency separation, i.e., ${N\times M}/{f_{\mathrm{range}}}$. Since FPS is constrained by the inverse of the measurement time *T*, it follows that:

$\mathrm{FPS}\leq{f_{\mathrm{range}}}/{N\times M}$ (S27)

This equation demonstrates that, for a fixed hardware configuration, the upper limit of FPS is inversely proportional to frame size. We observe that the acousto-optic modulation bandwidth $f_{\mathrm{range}}$ essentially represents the embodiment of SBP-T within the modulation framework, owing to their analogous multiplicative definitions towards frame size and FPS. In practice, to further avoid spectrum aliasing during the side-band detection, Supplementary Note 4 guides an exceptional frequency offset in between, resulting in a sacrifice of FPS for resolving frequencies optimally.

**Supplementary Note 14: Workflow of streamlined FACE-SPCM**

This section provides a detailed workflow for the streamlined FACE-SPCM system, as illustrated in Fig. S13. The system operates in an ultrafast, continuous mode, functioning without interruptions until a halt is initiated or the delivery buffer is exhausted. Utilizing the OFDM configuration for the FACE scheme with $\hat{f}_{\mathrm{shift}}=1,000\mathrm{Hz}$, the DAC (ATS9462, AlazarTech, configured with a sampling rate of 50 MSa s^-1^) requires 1 ms for data acquisition to perform single-shot reconstruction via FFT. This sets the upper limit of the refresh rate for the FACE-SPCM system at 1,000 Hz, encompassing both data acquisition and image reconstruction processes.

To ensure high quality in complex-field reconstruction or imaging through scattering media, a pre-processing calibration (as detailed in Supplementary Note 5) can be performed. The system is triggered in single-shot mode to capture an initial image, followed by a 1-ms data acquisition process. A blank image, without any target object, is established as the ground truth, requiring approximately 300 μs due to the efficiency of the FFT algorithm. The retrieval process involves an FFT operation (~282 μs) and an index extraction operation for display (~13 μs).


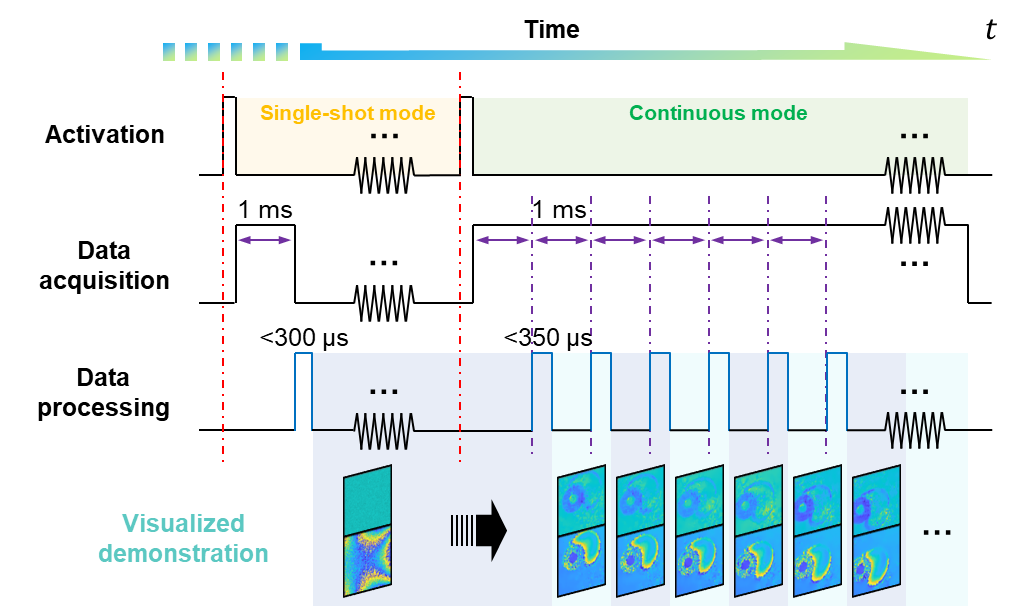


**Figure S13 | Workflow of the streamlined FACE-SPCM system.** The detailed procedure involves three main steps: activating acquisition mode, data acquisition, and data processing. A visual demonstration of the imaging process is provided for clarity.

After this initial calibration, the DAC switches to continuous mode for long-term data collection. As illustrated in Fig. S13, data signals are segmented into 1-ms intervals for continuous reconstruction. Each reconstruction process is triggered immediately following every 1-ms data collection. Since data acquisition and image reconstruction are performed independently and in parallel, subsequent reconstructions can proceed without delaying the next imaging cycle, provided the process remains within the 1-ms limit. Meanwhile, the visual demonstration at the bottom of the figure updates in real-time after each reconstruction. In continuous mode, image reconstruction takes slightly longer (<350 μs) than in single-shot mode due to additional calibration calculations. The data retrieval process includes the FFT operation (~282 μs), an additional calibration operation (~36 μs), and an index extraction operation for display (~13 μs).

**Reference**

1. E. Hahamovich, S. Monin, Y. Hazan et al., "Single pixel imaging at megahertz switching rates via cyclic Hadamard masks," Nature Communications **12**, 4516 (2021).

2. P. Kilcullen, T. Ozaki, and J. Liang, "Compressed ultrahigh-speed single-pixel imaging by swept aggregate patterns," Nature Communications **13**, 7879 (2022).

3. D. Wu, J. Luo, G. Huang et al., "Imaging biological tissue with high-throughput single-pixel compressive holography," Nature Communications **12**, 4712 (2021).

4. K. Goda, K. K. Tsia, and B. Jalali, "Serial time-encoded amplified imaging for real-time observation of fast dynamic phenomena," Nature **458**, 1145-1149 (2009).

5. J.-L. Wu, Y.-Q. Xu, J.-J. Xu et al., "Ultrafast laser-scanning time-stretch imaging at visible wavelengths," Light: Science & Applications **6**, e16196-e16196 (2017).

6. J. Teng, Q. Guo, M. Chen et al., "Time-encoded single-pixel 3D imaging," APL Photonics **5**, 020801 (2020).

7. E. D. Diebold, B. W. Buckley, D. R. Gossett et al., "Digitally synthesized beat frequency multiplexing for sub-millisecond fluorescence microscopy," Nature Photonics **7**, 806-810 (2013).

8. D. Schraivogel, T. M. Kuhn, B. Rauscher et al., "High-speed fluorescence image–enabled cell sorting," Science **375**, 315-320 (2022).

9. M. G. Moharam, and L. Young, "Criterion for Bragg and Raman-Nath diffraction regimes," Applied Optics **17**, 1757-1759 (1978).

10. S. Weinstein, and P. Ebert, "Data Transmission by Frequency-Division Multiplexing Using the Discrete Fourier Transform," IEEE Transactions on Communication Technology **19**, 628-634 (1971).

11. X. Wei, Y. Shen, J. C. Jing et al., "Real-time frequency-encoded spatiotemporal focusing through scattering media using a programmable 2D ultrafine optical frequency comb," Science Advances **6**, eaay1192 (2020).

12. C. Henry, "Theory of the linewidth of semiconductor lasers," IEEE Journal of Quantum Electronics **18**, 259-264 (1982).

13. A. Mussot, E. Lantz, H. Maillotte et al., "Spectral broadening of a partially coherent CW laser beam in single-mode optical fibers," Optics Express **12**, 2838-2843 (2004).
